# Transcriptome Analysis Identifies Key Genes Responsible for Red Coleoptiles in *Triticum Monococcum*

**DOI:** 10.3390/molecules24050932

**Published:** 2019-03-07

**Authors:** Dong Cao, Jiequn Fan, Xingyuan Xi, Yuan Zong, Dongxia Wang, Huaigang Zhang, Baolong Liu

**Affiliations:** 1 Qinghai Provincial Key Laboratory of Crop Molecular Breeding, Xining 810008, Qinghai, China; caodong@nwipb.cas.cn (D.C.); xixy@nwipb.cas.cn (X.X.); hgzhang@nwipb.cas.cn (H.Z.); 2 State Key Laboratory of Plateau Ecology and Agriculture, Qinghai University, Xining 800010, Qinghai, China; laughing1898@icloud.com (Y.Z.); wangdx1127@163.com (D.W.); 3 Shanghai Academy of Agricultural Sciences, Shanghai 201403, China; caod.08@163.com

**Keywords:** *T. monococcum*, coleoptile, anthocyanin biosynthesis, *MYB* transcription factor

## Abstract

Red coleoptiles can help crops to cope with adversity and the key genes that are responsible for this trait have previously been isolated from *Triticum aestivum*, *Triticum urartu*, and *Aegilops tauschii*. This report describes the use of transcriptome analysis to determine the candidate gene that controls the trait for white coleoptiles in *T. monococcum* by screening three cultivars with white coleoptiles and two with red coleoptiles. Fifteen structural genes and two transcription factors that are involved in anthocyanin biosynthesis were identified from the assembled UniGene database through BLAST analysis and their transcript levels were then compared in white and red coleoptiles. The majority of the structural genes reflected lower transcript levels in the white than in the red coleoptiles, which implied that transcription factors related to anthocyanin biosynthesis could be candidate genes. The transcript levels of *MYC* transcription factor *TmMYC-A1* were not significantly different between the white and red coleoptiles and all of the TmMYC-A1s contained complete functional domains. The deduced amino acid sequence of the MYB transcription factor TmMYB-A1 in red coleoptiles was homologous to TuMYB-A1, TaMYB-A1, TaMYB-B1, and TaMYB-D1, which control coleoptile color in corresponding species and contained the complete R2R3 MYB domain and the transactivation domain. TmMYB-a1 lost its two functional domains in white coleoptiles due to a single nucleotide deletion that caused premature termination at 13 bp after the initiation codon. Therefore, TmMYB-A1 is likely to be the candidate gene for the control of the red coleoptile trait, and its loss-of-function mutation leads to the white phenotype in *T. monococcum*.

## 1. Introduction

Red coleoptiles widely exist in wild relatives of cultivated wheat, such as *Triticum urartu*, *Aegilops tauschii*, and *T. monococcum*. Red coleoptiles, which are colored due to the accumulation of anthocyanin, can protect the emerging shoot from strong light, drought, and cold [1,2]. In addition, the coleoptile color trait is easy to observe and it has been employed as a useful characteristic in describing wheat varieties.

The anthocyanin biosynthetic pathway is relatively clear and the structural and regulatory genes have been isolated in several model plants [3,4,5,6]. There are some main structural genes in the anthocyanin biosynthesis pathway, including phenylalanine ammonia-lyase (PAL), cinnamate-4-hydroxylase (C4H), 4-coumarate: CoA ligase (4CL), shikimate O-hydroxycinnamoyltransferase (HCT), chalcone synthase (CHS), chalcone isomerase (CHI), flavanone 3-hydroxylase (F3H), flavonoid-3′-hydroxylase (F3′H), flavonoid-3′,5′-hydroxylase (F3′5′H), flavonol synthase (FLS), dihydrofavonol-4-reductase (DFR), leucoanthocyanidin dioxygenase (LDOX), flavonoid-3-O-glucosyltransferase (UFGT), anthocyanidin reductase (ANR), and leucoanthocyanidin reductase (LAR) [6,7]. A complex of transcription factors MYB and MYC with WD40 proteins regulate these structural genes in the main [8]. The MYB protein includes two incomplete repeats, R2-MYB and R3-MYB, and a transactivation domain, whereas the MYC protein contains bHLH-MYC_N, HLH, and ACT-like domains. The conserved R3 repeat of the MYB protein is involved in an interaction with MYC, which forms the complex that enables their transcriptional functionality [9,10,11]. Both structural and regulatory genes are crucial in anthocyanin biosynthesis, but allelic variation in the *MYB* and *MYC* genes causes color variations in different plant tissues [12,13,14,15,16]. The genes for MYC-type transcription factors that control anthocyanin-related traits are *TT8* in *Arabidopsis* [17], *AN1* in *Petunia* [18], *ThMYC4E* and *TaMYC1* in *T. aestivum* [15,19,20], and *AetMYC1* in *Ae. tauschii* [16]. A similar phenomenon has been observed for the genes of MYB-type transcription factors, including *MYB75*/*PAP1* and *AtMYB90*/*PAP2* in *Arabidopsis* [21], *AN2* in *Petunia* [22], *MYB1* in sweet potato [23], *LAP1* in legumes [24], *MdMYBA* and *MdMYB1* in apple [25,26], and *EsMYBA1* in *Epimedium sagittatum* [27].

In common wheat, the red coleoptile trait is controlled by *Rc1*, *Rc2*, and *Rc3*, which are located on the short arm of homeologous chromosomes 7A, 7B, and 7D [28]. *Rc1*, *Rc2*, and *Rc3* have recently been isolated and found to encode three MYB transcription factors—*TaMYB-A1*, *TaMYB-B1*, and *TaMYB-D1*—respectively [14,29,30]. The loss-of-function mutation of *TuMYB-A1*, which is homologous to *Rc1*, *Rc2*, and *Rc3*, resulted in the white coleoptile trait in *T. urartu* [31,32]. In *Ae. tauschii*, the allelic variation of the *MYC* gene *AetMYC1* caused coleoptile color differentiation [16]. Color variation also exists in the coleoptiles of *T. monococcum*, but the genetic mechanism has not been identified. High-throughput RNA sequencing (RNA-Seq) provides an economic and convenient approach for detecting novel transcripts, single nucleotide polymorphisms, small RNAs, and alternate splicing products, as well as sense and antisense transcripts [32,33]. The technique offers the advantage of measuring the gene expression levels and obtaining corresponding nucleotide sequences without reference sequences [34,35,36]. In this manuscript, transcriptomic analysis was conducted to identify the key gene that is associated with coleoptile color variation in *T. monococcum* by comparing the nucleotide sequence variation with the expression level of the structural genes and transcription factors that are associated with anthocyanin biosynthesis in red and white coleoptiles.

## 2. Results

### 2.1. High-Throughput RNA Sequencing

**Figure 1:**
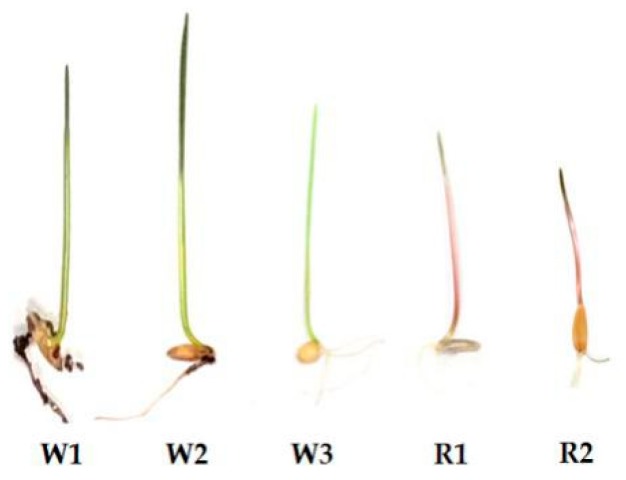
The coleoptiles of PI190939 (W1), CItr13961 (W2), CItr13962 (W3), PI554495 (R1), and PI554500 (R2).

To more comprehensively understand the coleoptile colour phenotype, the coleoptile colours of 143 *T. monococcum* cultivars were recorded. A total of 100 *T. monococcum* cultivars carried red coleoptiles and 43 carried white coleoptiles. The white coleoptile cultivars—PI190939 (W1), CItr13961 (W2), and CItr13962 (W3)—were selected to identify the key gene that is associated with the red coleoptile trait (Table S1; Figure 1), while two cultivars PI554495 (R1) and PI554500 (R2), carrying red coleoptiles, were chosen as the controls. Comparative transcriptome analysis was performed between the *T. monococcum* cultivars with red coleoptiles (R1 and R2) and white coleoptiles (W1, W2, and W3). After filtering, we obtained 53–60 million clean reads for each sample (Table S2). Trinity software was used to link the valid data to 294,658 UniGenes totaling 257,478,102 bp. The average length of each gene was 873 bp and the length of the N50 was 1466 bp.

**Figure 2:**
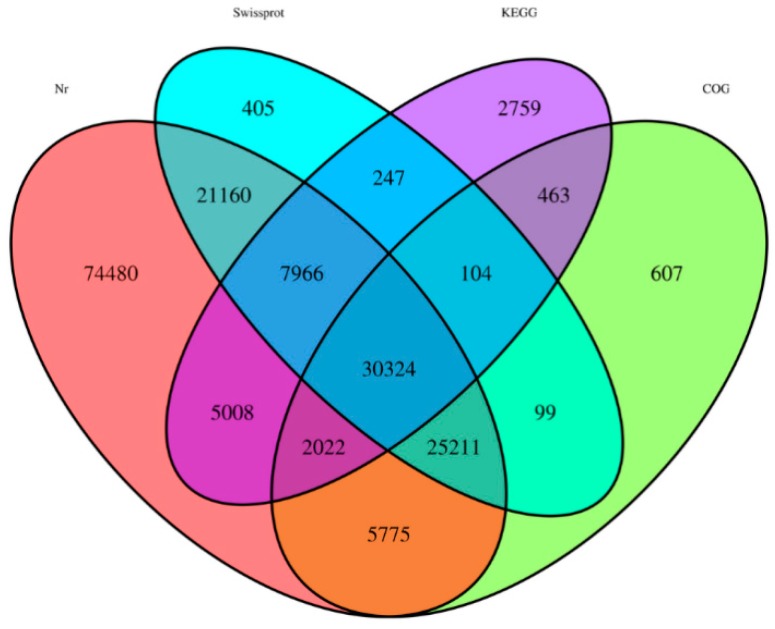
Outcome of the homology search of *T. monococcum* UniGenes.

BLASTX software was employed to annotate the assembled UniGenes using the non-redundant (Nr) (E_value ≦ 0.01), Swissprot (E_value ≦ 0.01), Kyoto Encyclopedia of Genes and Genomes (KEGG) (E_value ≦ 1e^−5^), and Clusters of Orthologous Groups (COG) (E_value ≦ 1e^−5^) databases. Totals of 171,946 (58.35%), 85,516 (29.02%), 48,893 (16.60), and 64,605 (21.93%) UniGenes could be annotated to the Nr, Swiss-Prot, KEGG, and COG databases, respectively (Figure 2; Table S3). A sum of 176,630 proteins was determined following the removal of repeated proteins.

### 2.2. Expression Profiles of Structural Genes Associated with Anthocyanin Biosynthesis

**Figure 3:**
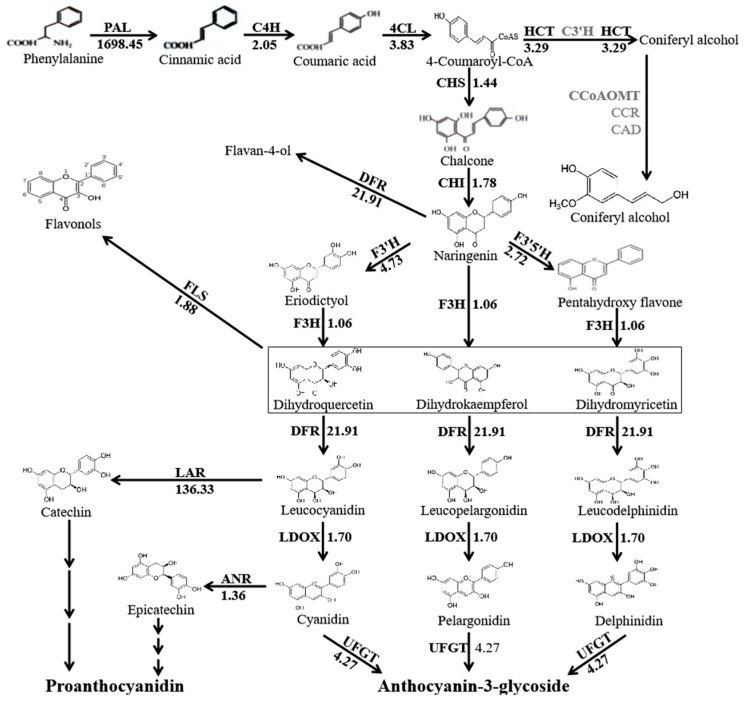
The expression level differences of anthocyanin biosynthesis-related structural genes in red and white coleoptiles. Arrows show the metabolic stream; the light color indicates that these genes are in the assembled UniGenes; the numbers represent the increased level of expression in red coleoptiles compared with white coleoptiles.

Coleoptile color variation is associated with the biosynthesis of anthocyanin and 15 structural genes that are related to anthocyanin biosynthesis derived from *Zea mays* were selected for a BLAST search of the assembled UniGene database and NCBI database. A total of 36 UniGenes were homologous to *PAL*, *C4H*, *4CL*, *HCT*, *CHS*, *CHI*, *F3H*, *FLS*, *F3′H*, *F3′5′H*, *DFR*, *LAR, LDOX*, *ANR*, and *UFGT* (Figure 3). The only UniGene that could not be identified was that homologous to *C3′H*. The phylogenetic trees were constructed using the deduced amino acid sequences of 36 unigenes in *T. monococcum* and structural genes associated with anthocyanin biosynthesis in other species (Figures S1–S15). The same structural genes were assigned to the same branch (Figures S1–S15). The fragments per kilobase of transcript per million mapped reads (FPKM) values of these genes were determined in order to evaluate their relative expression levels. The anthocyanin biosynthesis pathway was exhibited in *T. monococcum*, as described by Holton and Cornish [37] and Winkel-Shirley [6] (Figure 3). All of the structural genes exhibited higher expression levels in red coleoptiles (Figure 3; Table S4). The relative transcript level is between 1.06 and 1698.45 times higher in red coleoptiles than white coleoptiles, which implied that the transcription factor might be the key gene for the white coleoptile trait in *T. monococcum*.

### 2.3. Sequence Characteristics and Expression Profiles of Regulatory Genes Associated with Anthocyanin Biosynthesis

*TuMYB-A1*, *TaMYB-A1*, *TaMYB-B1*, and *TaMYB-D1* control thecoleoptile color variation in *T. urartu* and *T. aestivum*, respectively [29,30,31], while *AetMYC1* is associated with variation of this trait in *Ae. Tauschii* [16]. Therefore, *TuMYB-A1* and *AetMYC1* were used in a BLAST search to identify homologous genes from the assembled UniGene database. One MYB transcription factor UniGenes (*R1.29805_c0_g6_i1*) and three MYC transcription factors (W2.26064_c0_g1_i6, W2.26064_c0_g1_i7, and W2.26064_c0_g1_ i12) UniGenes were obtained with scores >100. Each MYC contained between one and eight transcripts. A phylogenetic tree was constructed with the neighbor-joining method using the deduced amino acid sequences of the three MYC transcription factors and other MYCs that are involved in anthocyanin biosynthesis (Figure S16). W2.26064_c0_g1_i6, W2.26064_c0_g1_i7, and W2.26064_c0_g1_ i12 were assigned to the same branch as AetMYC1 (Figure S1), the key gene that is related to red coleoptiles in *T. aestivum* and *Ae. tauschii* [16,29]. W2.26064_c0_g1_i6, W2.26064_c0_g1_i7, and W2.26064_c0_g1_ i12 were splicing transcripts from the same gene, which was named *TmMYC1*. When compared with white coleoptiles, the average transcript levels of *TmMYC1* were lower in red coleoptiles (Table S4). The deduced amino acid sequences of TmMYC-A1 from white and red coleoptiles were the same length and they contained the same primary function domains, including the integrated bHLH-MYC_N domain, which is essential for protein–protein interaction, the HLH domain, which can facilitate protein-DNA binding, and the ACT-like domain, which initiates transcription (Figure S17).

**Figure 4:**
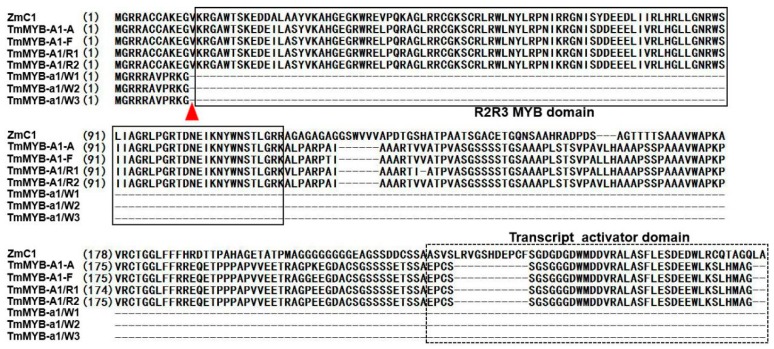
The deduced amino acid sequence alignment of TmMYB-A1 from R1, R2, W1, W2, W3, and MYBs, which regulate anthocyanin biosynthesis in other species. The solid frame represents the R2R3 MYB domain and the dotted frame represents the transcript activator domain. The red triangle represents the location of the stop codon.

The phylogenetic tree was also constructed by using the deduced amino acid sequences of the UniGenes *R1.29805_c0_g6_i1* and other MYBs that were involved in anthocyanin biosynthesis (Figure S18). The MYB transcription factor UniGene R1.29805_c0_g6_i1 was assigned to the same branch as TuMYB-A1, TaMYB-A1, TaMYB-B1, and TaMYB-D1 (Figure S3), which control coleoptile color in *T. urartu* [31] and *T. aestivum* [29,30,38]. *R1.29805_c0_g6_i1* was named *TmMYB-A1*. The average transcript levels of *TmMYB-A1* in red coleoptiles were 13.31 times greater than in white coleoptiles. All *TmMYB-a1* from white coleoptiles exhibited one base deletion (G) at 13 bp when compared with *TmMYB-A1* from red coleoptiles. The deduced amino acid sequences of TmMYB-A1 from the red coleoptiles contained the primary functional structure of the MYB protein R2R3 MYB and transcript activator domains (Figure 4), while TmMYB-a1 lost the R2R3 MYB and transcript activator domains, because the frame shift mutation caused premature termination of translation for the encoded protein (Figure 4).

## 3. Discussion

The comparative transcriptome analysis demonstrated that the structural genes involved in anthocyanin biosynthesis showed lower transcript abundance in white coleoptiles than the red coleoptiles, and these structural genes are primarily regulated by the MYB-bHLH-WD40 complex [39]. Loss of function or low expression of a *MYB* or *MYC* transcription factors could impede anthocyanin biosynthesis in coleoptiles [16,40,41,42] and, hence, the transcription factor is likely to be the candidate gene for coleoptile color variation in *T. monococcum*.

The MYC transcription factor *TmMYC1* was homologous to *AeMYC1*, which controls color variation in *Ae. tauschii*. However, the transcript levels of *TmMYC1* showed no significant difference between the white and red coleoptiles. All of the deduced amino acid sequences of *TmMYC1* from R1, R2, W1, W2, and W3 contained the integrated bHLH-MYC_N domain, HLH domain, and ACT-like domain. This suggested the *MYC* transcription factor could not be the key gene for the white coleoptile trait in *T. monococcum*. With regard to the MYB transcription factor, *TmMYB-A1* was on the same branch as TuMYB-A1, TaMYB-A1, TaMYB-B1, and TaMYB-D1 (Figure S18), which control coleoptile color in *T. urartu* [31] and *T. aestivum* [29,30,38] and the transcript levels of *TmMYB-A1* in red coleoptiles was significantly higher than in the white coleoptiles. The deduced amino acid sequence of TmMYB-A1 was homologous to TuMYB-A1 and it contained the complete R2R3 MYB domain and transcript activator domain, while the deduced amino acid sequences of TmMYB-a1 had lost the R2R3 MYB domain and transcript activator domain, because a single nucleotide deletion caused premature termination. Functional loss of its homologous genes, TuMYB-A1, TaMTB7A, TaMTB7B, and TaMTB7D, produced the white coleoptile in *T. urartu* [31] and *T. aestivum* [29,30,38] and, therefore, *TmMYB-a1* appeared to be the key gene that is responsible for the white coleoptile trait in *T. monococcum* accessions W1, W2, and W3, as signified by low transcript levels and the loss of function.

## 4. Materials and Methods

### 4.1. Plant Materials

A total of 143 *T. monococcum* cultivars were obtained from the USDA-ARS National Small Grains Collection [43] and the Institute of Genetics and Development Biology, Chinese Academy of Sciences (Table S4). Ten seeds were germinated to survey coleoptile color [30].

### 4.2. Preparation of Total RNA and cDNA

Total RNA was isolated from the coleoptiles of five-day-old seedlings using the TIANGEN RNAprep Pure Plant Kit (Tiangen Company, Beijing, China). The total RNA of each sample was quantified and qualified with an Agilent 2100 Bioanalyzer (Agilent Technologies, Palo Alto, CA, USA), NanoDrop (Thermo Fisher Scientific, Wilmington, DE, USA), and 1% agrose gel. cDNA was obtained from the total RNA using the Thermo RevertAid First Strand cDNA Synthesis Kit (Thermo Fisher Scientific, St. Louis, MO, USA).

### 4.3. Transcriptome Analysis

Ten cDNA library preparations were constructed (two replicates per accession) according to the manufacturer’s protocol for mRNA-Seq sample preparation (Illumina, Inc., San Diego, CA, USA). Ten cDNA library products were sequenced by Illumina paired-end sequencing technology, with read lengths of 150 bp on the Illumina HiSeq X instrument by Genewiz Biotechnology Co. Ltd. (Suzhou, China). The raw sequence reads were stored in the National Center for Biotechnology Information (NCBI) SRA database, with the accession number SUB5053734. Before assembly, the raw paired-end reads were filtered in order to obtain high quality clean reads. Low quality sequences were removed, including the sequences with ambiguous bases (denoted by more than 10% “N” in the sequence trace), low quality reads (the rate of reads in which a quality value ≤10 is greater than 20%), and reads with adaptors. After filtering, the high quality reads were assembled by Trinity, with default parameters, to construct unique consensus sequences that are based on the sequences from the *T. monococcum* accessions R1, R2, W1, W2, and W3 genotypes [44]. UniGenes that were differentially expressed between red and white coleoptile genotypes were analyzed using chi-square tests with IDEG6 software (University of Padua, Padua, Italy) [45]. The UniGene expression level was calculated while using the FPKM values. The false discovery rate (FDR) method was introduced to determine the threshold p-value at FDR ≤ 0.05, and the absolute value of |log2Ratio| ≥ 2 was used as the threshold to determine the significance of the differential expression of the UniGenes. The genes that are related to anthocyanin biosynthesis in KEGG pathways [46] were collected and then aligned to the UniGenes from a transcriptome mixture of red and white coleoptiles using the BLASTX algorithm with an E-value of < 1.0 × 10^−5^.

### 4.4. Bioinformatic Analysis

The sequence alignments were conducted using the Vector NTI 10 software package (Thermo Fisher Scientific). MEGA5 software was used to construct the phylogenetic tree (Tokyo Metropolitan University, Hachioji, Tokyo, Japan) using the neighbor-joining method [47].

## 5. Conclusions

In this study, transcriptome analysis demonstrated that structural genes showed lower transcript levels in the white than the red coleoptiles in *T. monococcum*, which implied that a transcription factor was likely to be the key gene for determining white-colored coleoptiles. The transcript levels of *MYC* transcription factor *TmMYC-A1* were not significantly different between the white and red coleoptiles and all TmMYC-A1s contained the complete functional domains in white and red coleoptiles. The MYB transcription factor TmMYB-A1, homologous to genes controlling coleoptile color in common wheat, contained the complete functional domain in the red coleoptiles, while TmMYB-a1 had lost the functional domains in white coleoptiles due to a frameshift mutation. Moreover, the transcript levels of *TmMYB-A1* in red coleoptiles were significantly higher than in white coleoptiles. Therefore, *TmMYB-a1* is likely to be the key gene that is responsible for the white coleoptile trait in *T. monococcum,* as suggested by the low levels of transcription and functional loss.

## Supplementary Materials

The following are available online: Figure S1. Phylogenetic relationships of the deduced amino acid sequences of one PAL and other PAL involved in anthocyanin biosynthesis; Figure S2. Phylogenetic relationships of the deduced amino acid sequences of three 4CL and other 4CL involved in anthocyanin biosynthesis; Figure S3. Phylogenetic relationships of the deduced amino acid sequences of four ANR and other ANR involved in anthocyanin biosynthesis; Figure S4. Phylogenetic relationships of the deduced amino acid sequences of two C4H and other C4H involved in anthocyanin biosynthesis; Figure S5. Phylogenetic relationships of the deduced amino acid sequences of three CHI and other CHI involved in anthocyanin biosynthesis; Figure S6. Phylogenetic relationships of the deduced amino acid sequences of two CHS and other CHS involved in anthocyanin biosynthesis; Figure S7. Phylogenetic relationships of the deduced amino acid sequences of four DFR and other DFR involved in anthocyanin biosynthesis; Figure S8. Phylogenetic relationships of the deduced amino acid sequences of one F3′5′H and other F3′5′H involved in anthocyanin biosynthesis; Figure S9. Phylogenetic relationships of the deduced amino acid sequences of two F3H and other F3H involved in anthocyanin biosynthesis; Figure S10. Phylogenetic relationships of the deduced amino acid sequences of one F3′H and other F3′H involved in anthocyanin biosynthesis; Figure S11. Phylogenetic relationships of the deduced amino acid sequences of one HCT and other HCT involved in anthocyanin biosynthesis; Figure S12. Phylogenetic relationships of the deduced amino acid sequences of two LAR and other LAR involved in anthocyanin biosynthesis; Figure S13. Phylogenetic relationships of the deduced amino acid sequences of two LDOX and other LDOX involved in anthocyanin biosynthesis; Figure S14. Phylogenetic relationships of the deduced amino acid sequences of three UFGT and other UFGT involved in anthocyanin biosynthesis; Figure S15. Phylogenetic relationships of the deduced amino acid sequences of two FLS and other FLS involved in anthocyanin biosynthesis; Figure S16. Phylogenetic relationships of the deduced amino acid sequences of 3 MYCs and other MYCs involved in anthocyanin biosynthesis; Figure S17. Amino acid sequence alignment of MYC transcription factors from R1, R2, W1, W2, W3, and other species; Figure S18. Phylogenetic relationships between MYBs from R1, R2, W1, W2, W3, and other species; Table S1. The information of *T. monococcum* used in this study; Table S2. Summary of sequencing data; Table S3. The annotation summary of predicted protein numbers from various databases; Table S4. Information on unigenes associated with anthocyanin biosynthesis in coleoptiles.
