# Supplementary material for: Transcriptome Analysis Identifies Key Genes Responsible for Red Coleoptiles in Triticum Monococcum
Source: Molecules. 2019 Mar 7;24(5):932. doi: 10.3390/molecules24050932 (PMC6429503; doi:10.3390/molecules24050932)
Supplement: Supplementary file 1 [file molecules-24-00932-s001.pdf]

Article

Transcriptome Analysis Identifies the Key Genes Responsible for The White Coleoptile Trait in *Triticum monococcum*

Dong Cao[1,2,†], Jiequn Fan[3,†], Xingyuan Xi[1], Yuan Zong[2], Dongxia Wang[2], Huaigang Zhang[1], Baolong Liu[1]

[1] Qinghai Provincial Key Laboratory of Crop Molecular Breeding, Xining 810008, Qinghai, China; caodong@nwipb.cas.cn (D.C.); xyxi@nwipb.cas.cn (X.Y.); hgzhang@nwipb.cas.cn (H.G.)

[2] State Key Laboratory of Plateau Ecology and Agriculture, Qinghai University, Qinghai, Xining 800010, China; laughing1898@icloud.com (Y.Z.); wangdx1127@163.com (D.X.)

[3] Shanghai Academy of Agricultural Sciences, Shanghai 201403, China

* Correspondence: blliu@nwipb.cas.cn (B.L.) Tel.: +86-971-6143-610 (B.L.) Fax: +86-971-6143-282 (B.L.)

† These authors contributed equally to this paper.

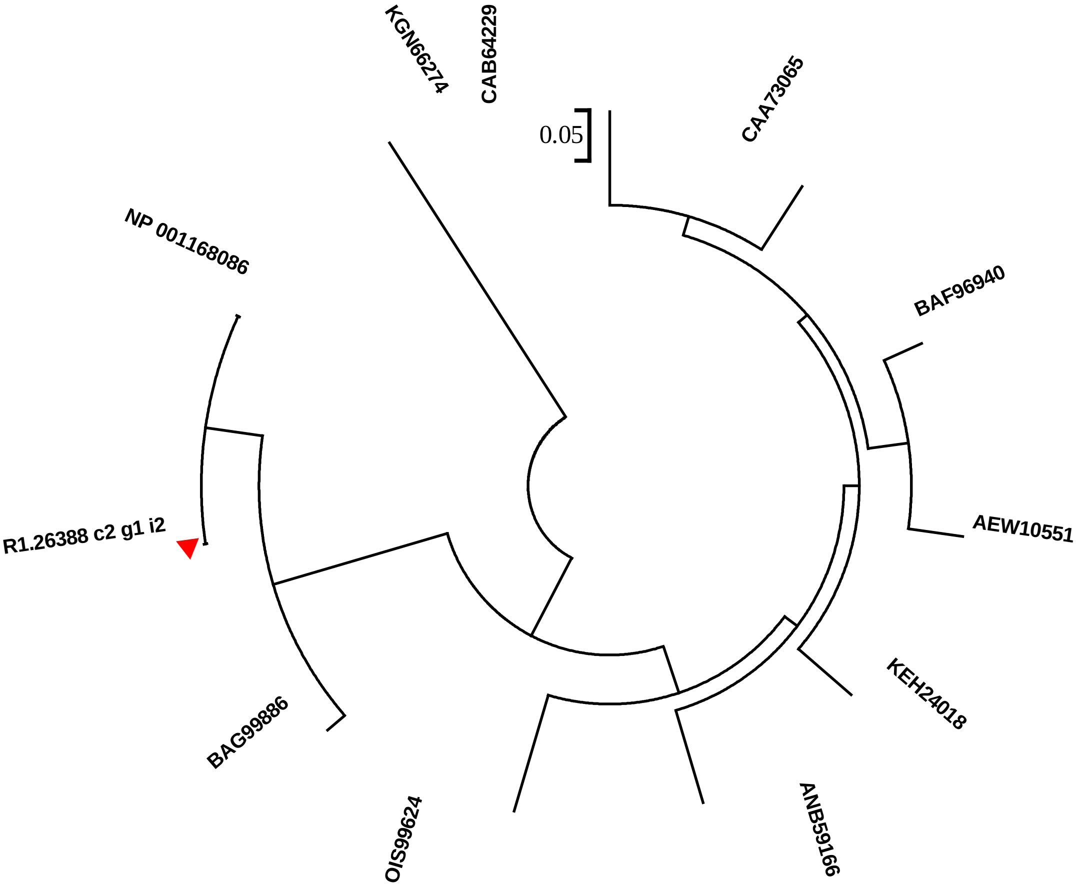

Figure S1. Phylogenetic relationships of the deduced amino acid sequences of one PAL and other PAL involved in anthocyanin biosynthesis. The accession numbers of these proteins are as follows: CAB64229: Arabidopsis thaliana/PAL; KGN66274: Cucumis sativus/PAL; CAA73065: Helianthus annuus/PAL; KEH24018.1: Medicago truncatula/PAL; OIS99624: Nicotiana attenuata/PAL; BAF96940: Nicotiana tabacum/PAL; BAG99886: Oryza sativa/PAL; AEW10551: Solanum lycopersicum/PAL; ANB59166: Vitis vinifera/PAL; NP_001168086: Zea mays/PAL.

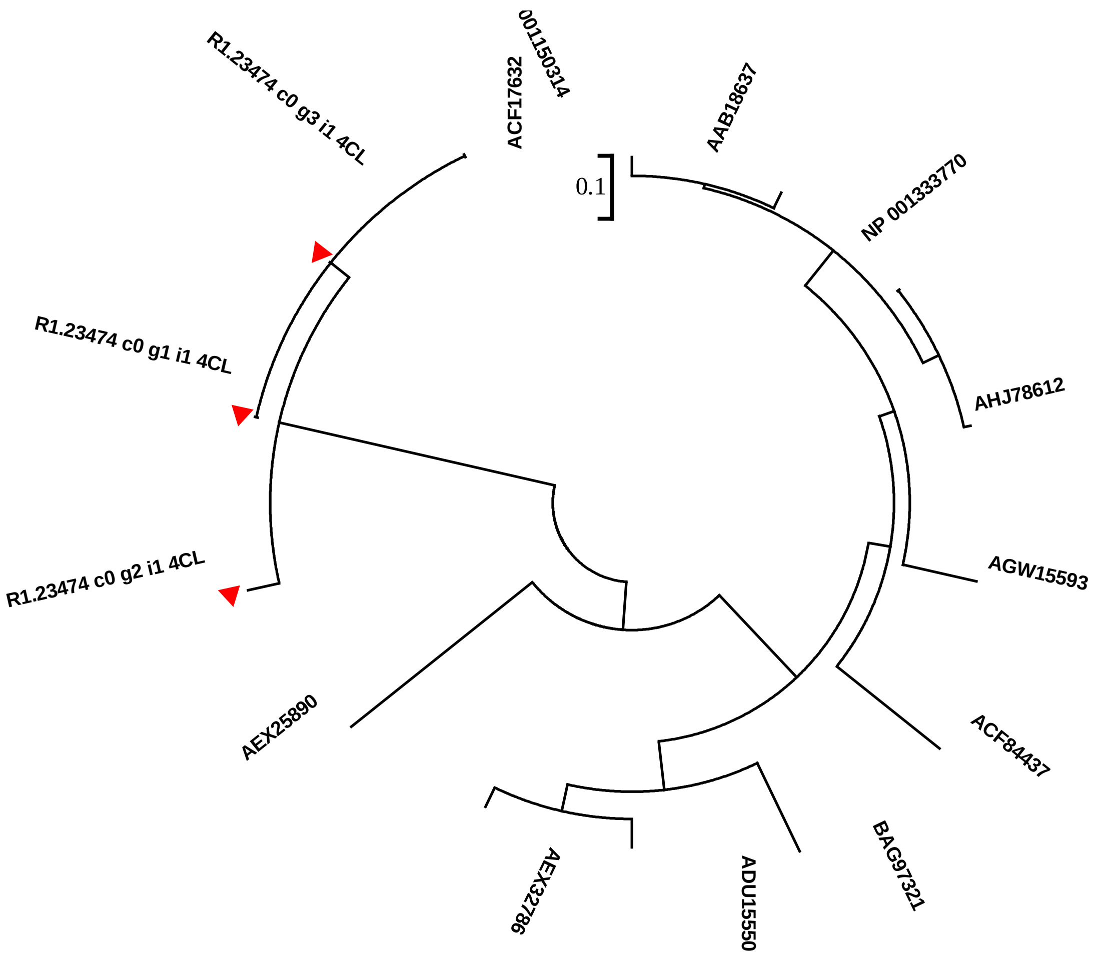

Figure S2. Phylogenetic relationships of the deduced amino acid sequences of three 4CL and other 4CL involved in anthocyanin biosynthesis. The accession numbers of these proteins are as follows: NP_001150314: Zea mays/4CL; ACF17632: Capsicum annuum/4CL; AEX25890: Glycine max/4CL; ADU15550: Gossypium hirsutum/4CL; AAB18637: Nicotiana tabacum/4CL; BAG97321: Oryza sativa/4CL; AGW15593: Pyrus x bretschneideri/4CL; NP_001333770: Solanum lycopersicum/4CL; AHJ78612: Solanum tuberosum/4CL; AEX32786: Vitis vinifera/4CL; ACF84437: Zea mays/4CL.

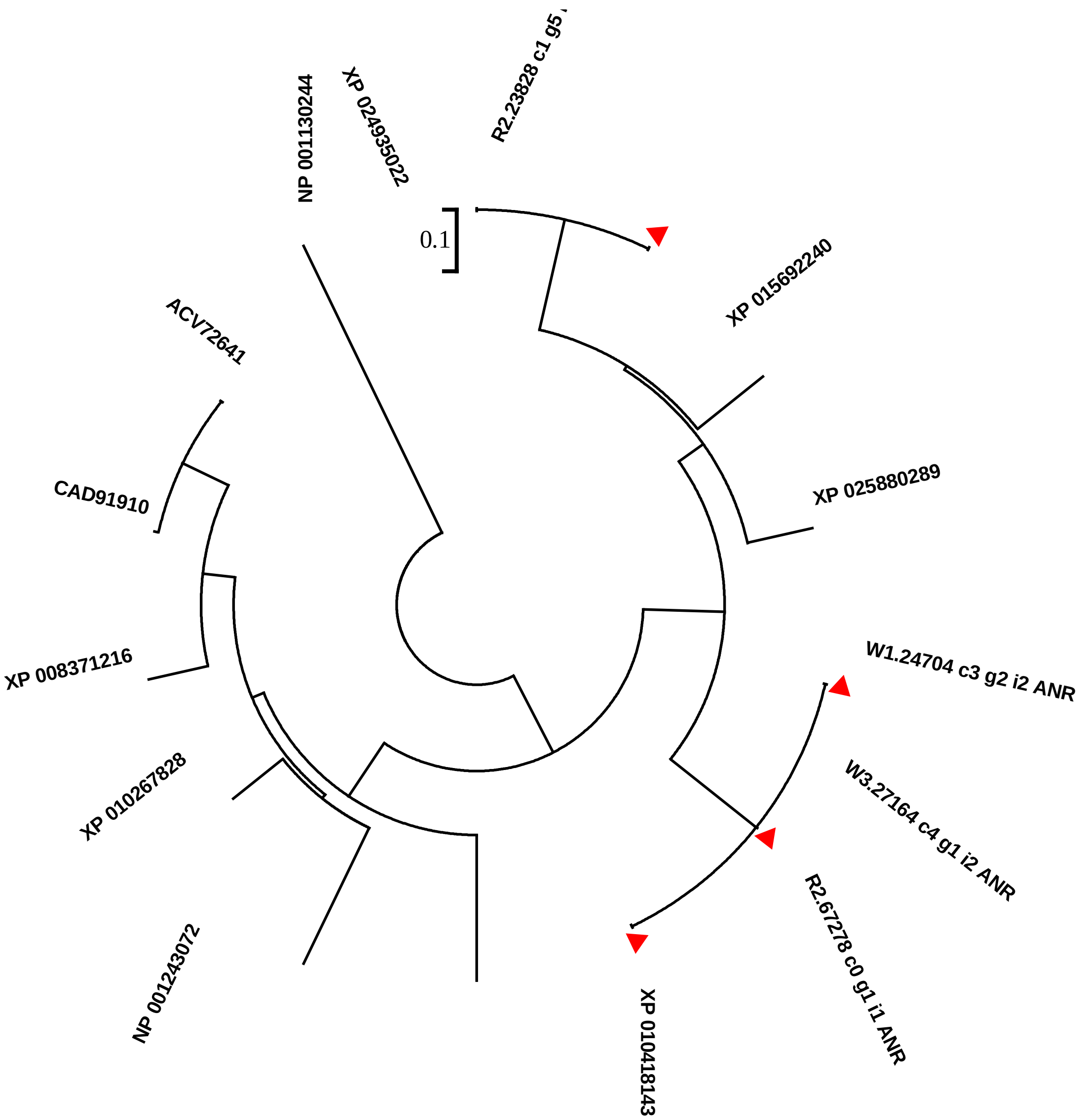

Figure S3. Phylogenetic relationships of the deduced amino acid sequences of four ANR and other ANR involved in anthocyanin biosynthesis. The accession numbers of these proteins are as follows: XP_010418143: Camelina sativa/ANR; NP_001243072: Glycine max/ANR; CAD91910: Gossypium arboreum/ANR; ACV72641: Gossypium hirsutum/ANR; XP_008371216: Malus domestica/ANR; XP_010267828: Nelumbo nucifera/ANR; XP_015692240: Oryza brachyantha/ANR; XP_025880289: Oryza sativa/ANR; XP_024935022: Ziziphus jujuba/ANR; NP_001130244: Zea mays/ANR.

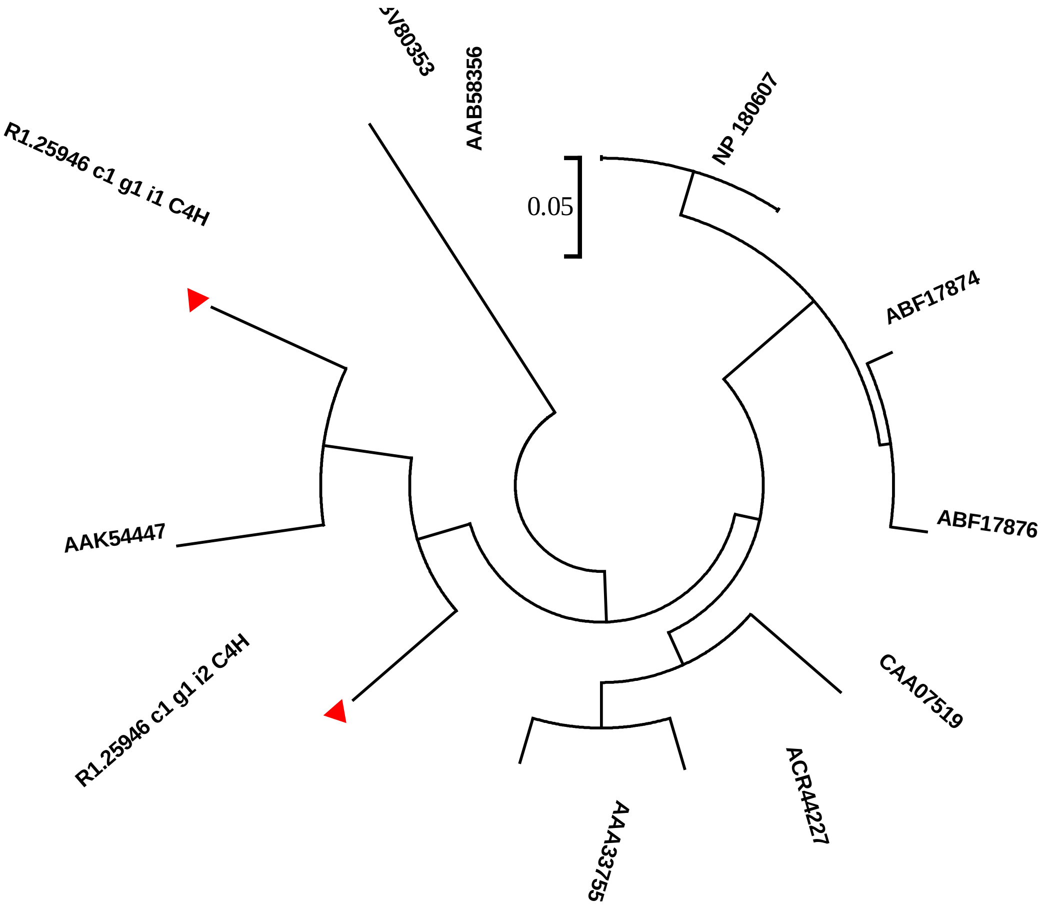

Figure S4. Phylogenetic relationships of the deduced amino acid sequences of two C4H and other C4H involved in anthocyanin biosynthesis. The accession numbers of these proteins are as follows: AAB58356: Arabidopsis thaliana/C4H; ABF17874: Brassica napus/C4H; ABF17876: Brassica napus/C4H; CAA07519: Cicer arietinum/C4H; ACR44227: Glycine max/C4H; ABV80353: Selaginella moellendorffii/C4H; AAK54447: Sorghum bicolor/C4H; AAA33755: Vigna radiata/C4H; NP_180607: Zea mays/C4H.

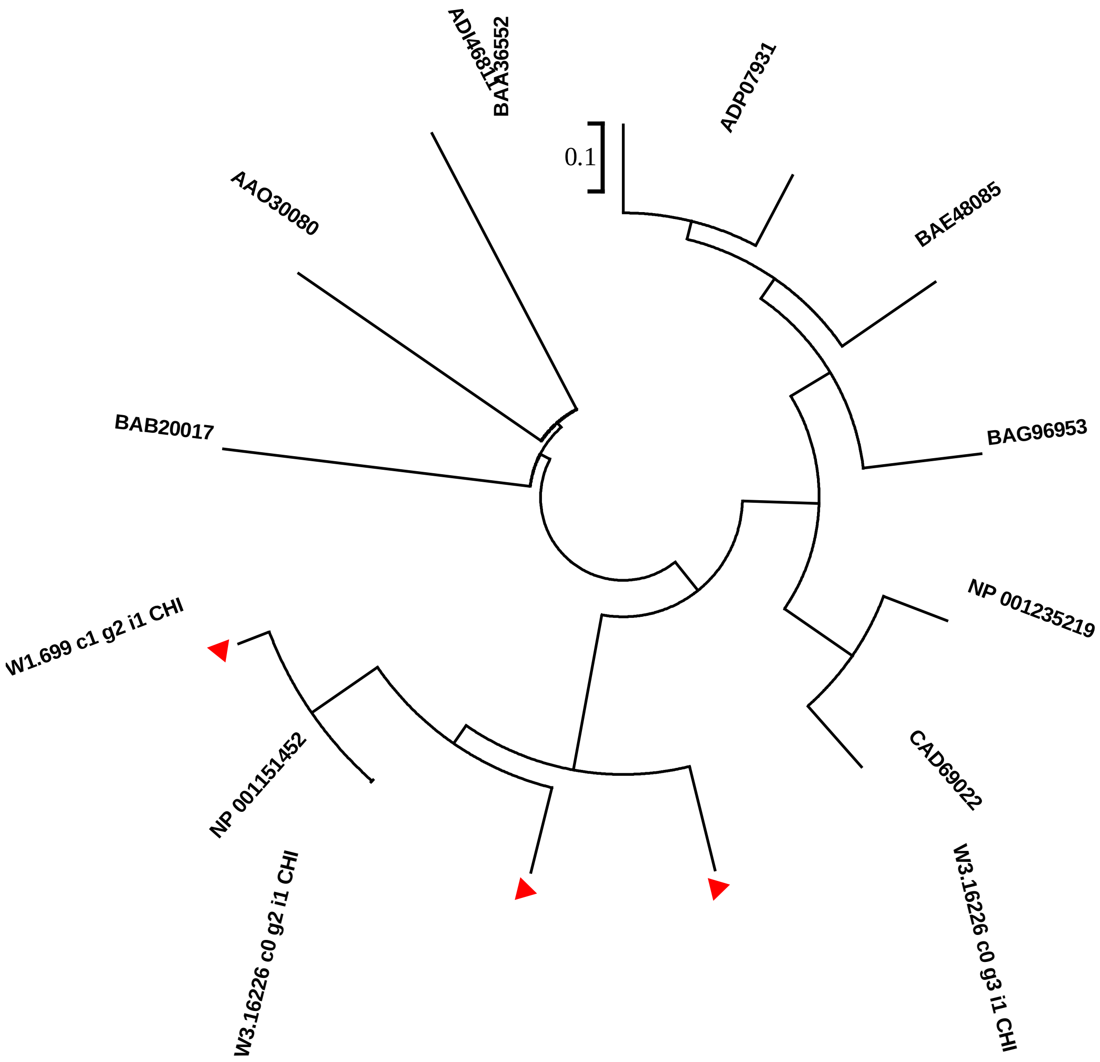

Figure S5. Phylogenetic relationships of the deduced amino acid sequences of three CHI and other CHI involved in anthocyanin biosynthesis. The accession numbers of these proteins are as follows: AAO30080: Arabidopsis thaliana/CHI; BAA36552: Citrus sinensis/CHI; NP_001235219: Glycine max/CHI; CAD69022: Lotus japonicus/CHI; BAE48085: Nicotiana tabacum/CHI; BAG96953: Oryza sativa/CHI; ADP07931: Vitis vinifera/CHI; NP_001151452: Zea mays/CHI.

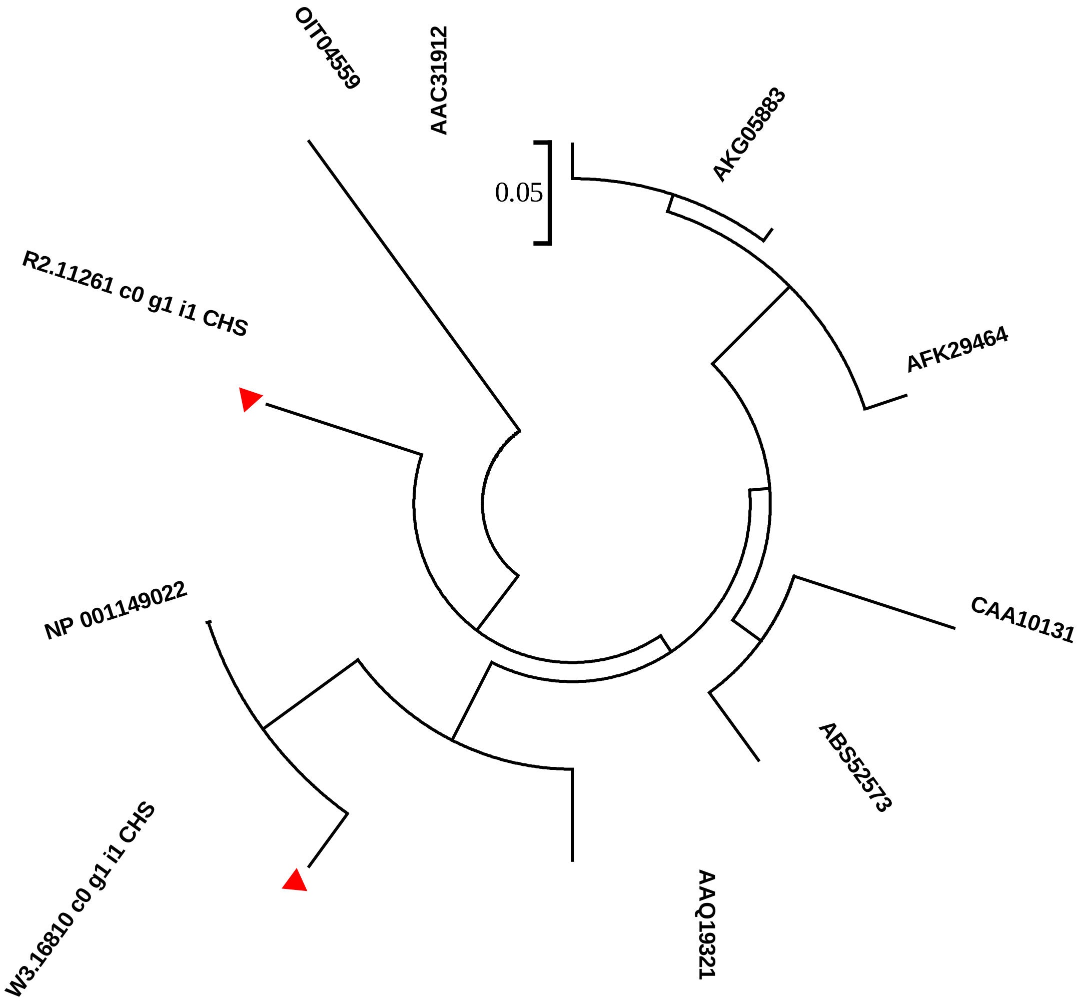

Figure S6. Phylogenetic relationships of the deduced amino acid sequences of two CHS and other CHS involved in anthocyanin biosynthesis. The accession numbers of these proteins are as follows: AFK29464: Arabidopsis lyrata/CHS; AAC31912: Brassica napus/CHS; CAA10131: Cicer arietinum/CHS; AKG05883: Eutrema salsugineum/CHS; ABS52573: Gossypium hirsutum/CHS; OIT04559: Nicotiana attenuata/CHS; AAQ19321: Triticum aestivum/CHS; NP_001149022: Zea mays/CHS.

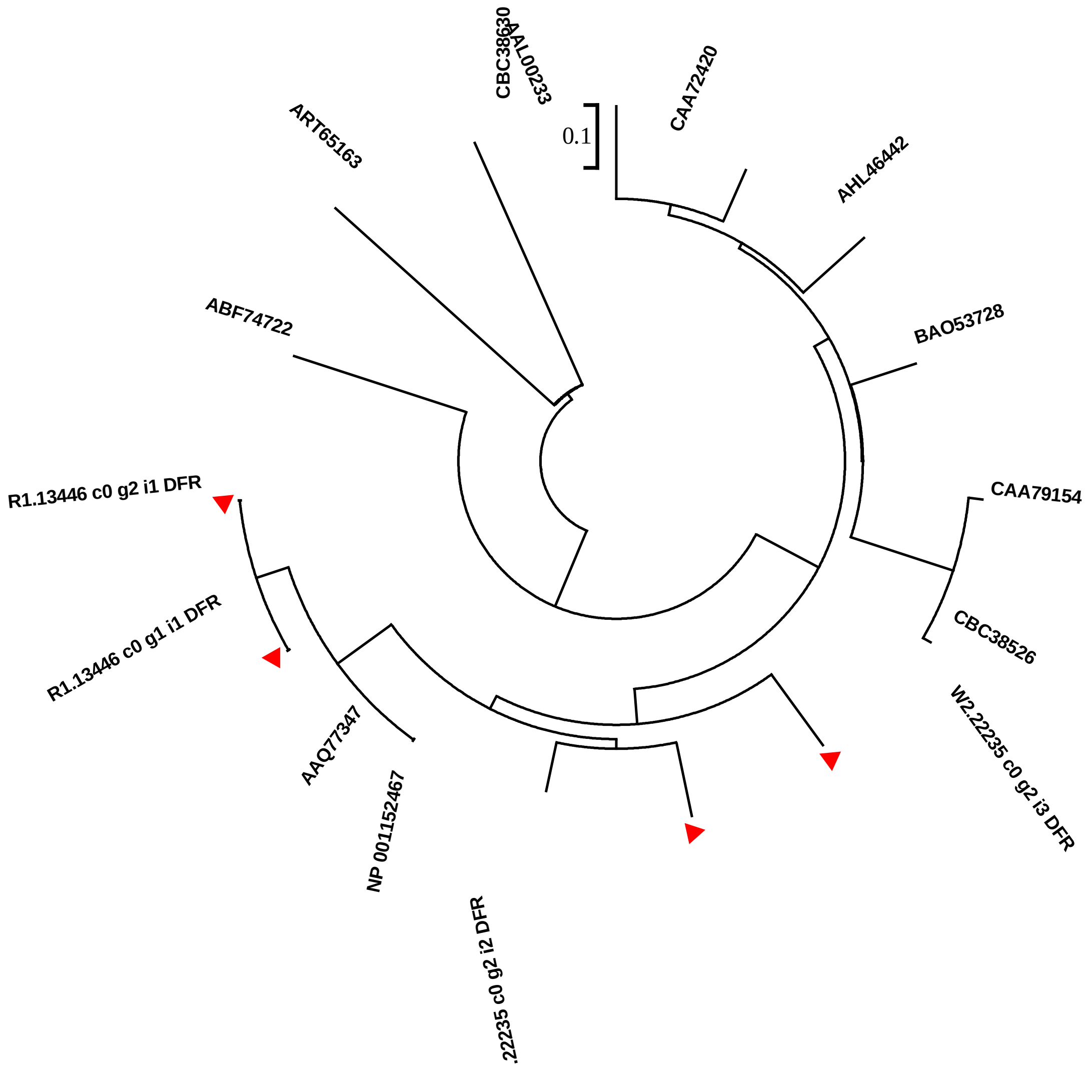

Figure S7. Phylogenetic relationships of the deduced amino acid sequences of four DFR and other DFR involved in anthocyanin biosynthesis. The accession numbers of these proteins are as follows: CBC38630: Arabidopsis thaliana/DFR; ABF74722: Arabidopsis thaliana/DFR; AHL46442: Fragaria vesca/DFR; BAO53728: Glycine max/DFR; ART65163: Gracilaria changii/DFR; CAA79154: Solanum lycopersicum/DFR; CBC38526: Solanum tuberosum/DFR; AAL00233: Streptococcus pneumoniae/DFR; AAQ77347: Triticum aestivum/DFR; CAA72420: Vitis vinifera/DFR; NP_001152467: Zea mays/DFR.

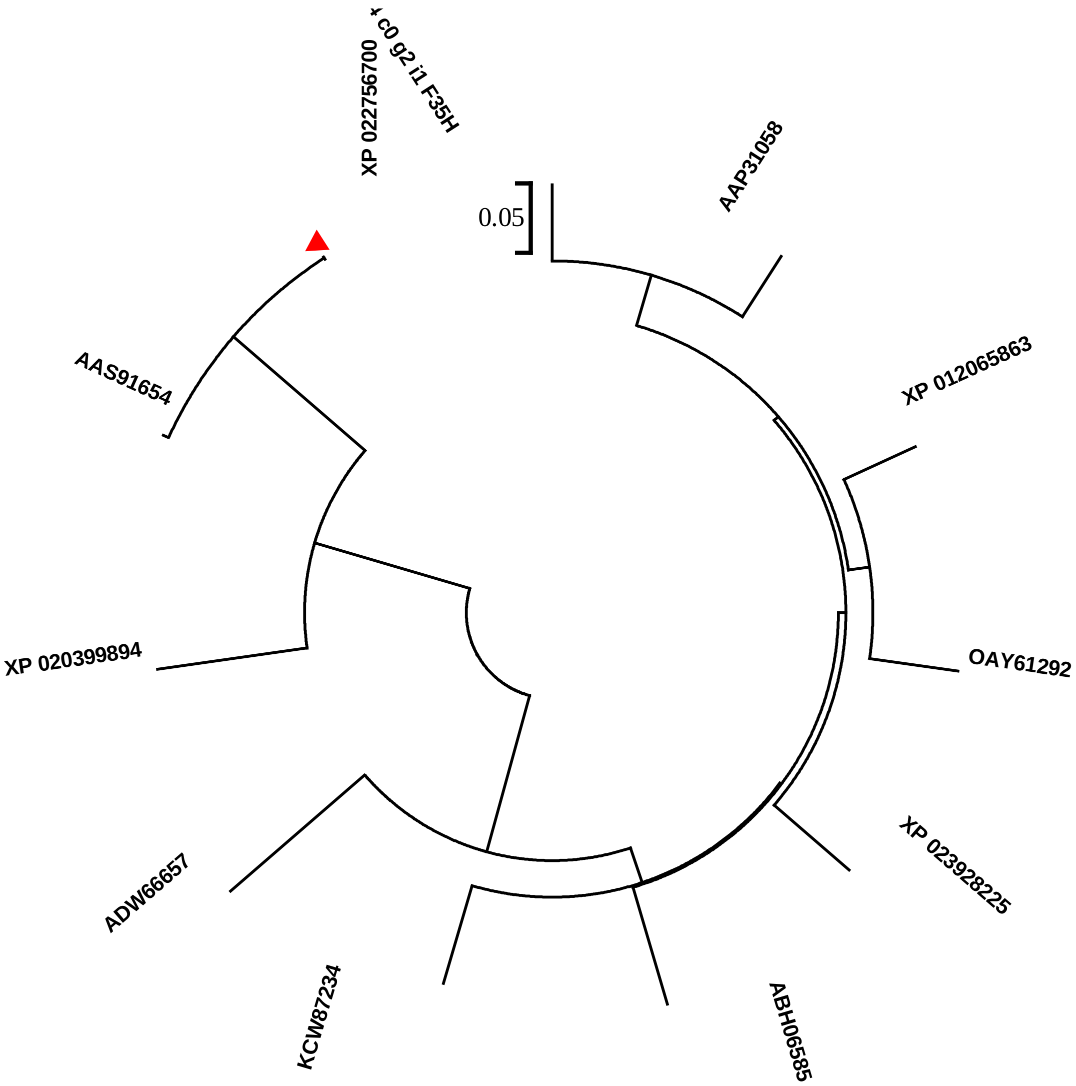

Figure S8. Phylogenetic relationships of the deduced amino acid sequences of one F3'5'H and other F3'5'H involved in anthocyanin biosynthesis. The accession numbers of these proteins are as follows: XP_022756700: Durio zibethinus/F3'5'H; KCW87234: Eucalyptus grandis/ F3'5'H; AAP31058: Gossypium hirsutum/F3'5'H; XP_012065863: Jatropha curcas/F3'5'H; OAY61292: Manihot esculenta/F3'5'H; XP_023928225: Quercus suber/F3'5'H; ADW66657: Solanum tuberosum/F3'5'H; AAS91654: Triticum aestivum/ F3'5'H; ABH06585: Vitis vinifera/ F3'5'H; XP_020399894: Zea mays/ F3'5'H.

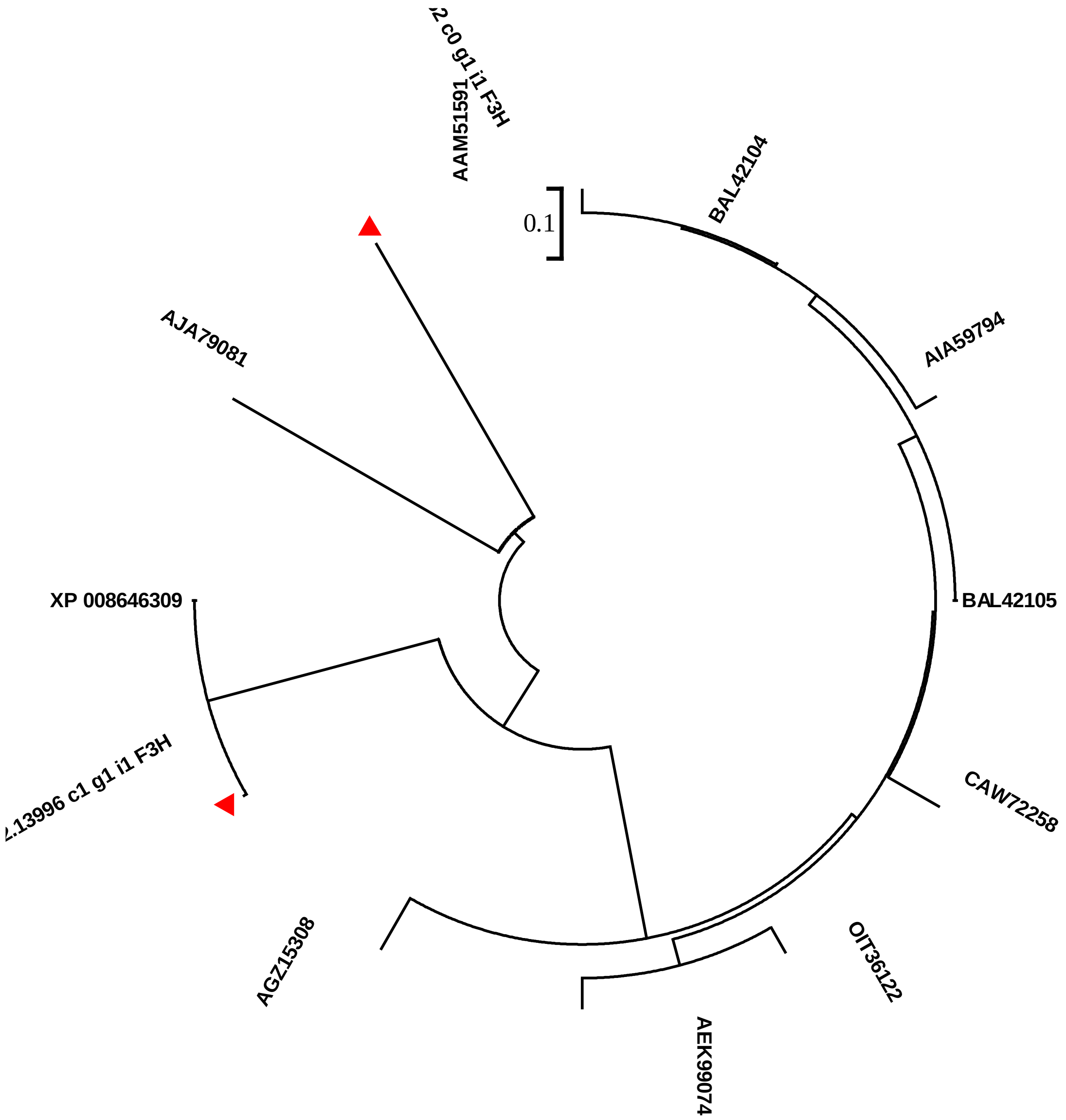

Figure S9. Phylogenetic relationships of the deduced amino acid sequences of two F3H and other F3H involved in anthocyanin biosynthesis. The accession numbers of these proteins are as follows: AAM51591: Arabidopsis thaliana/F3H; AIA59794: Brassica napus/F3H; BAL42105: Cardamine glauca/F3H; BAL42104: Cardamine resedifolia/F3H; CAW72258: Glycine max/F3H; OIT36122: Nicotiana attenuata/F3H; AJA79081: Prunus persica/F3H; AGZ15308: Pyrus x bretschneideri/F3H; AEK99074: Solanum lycopersicum/F3H; XP_008646309: Zea mays/F3H.

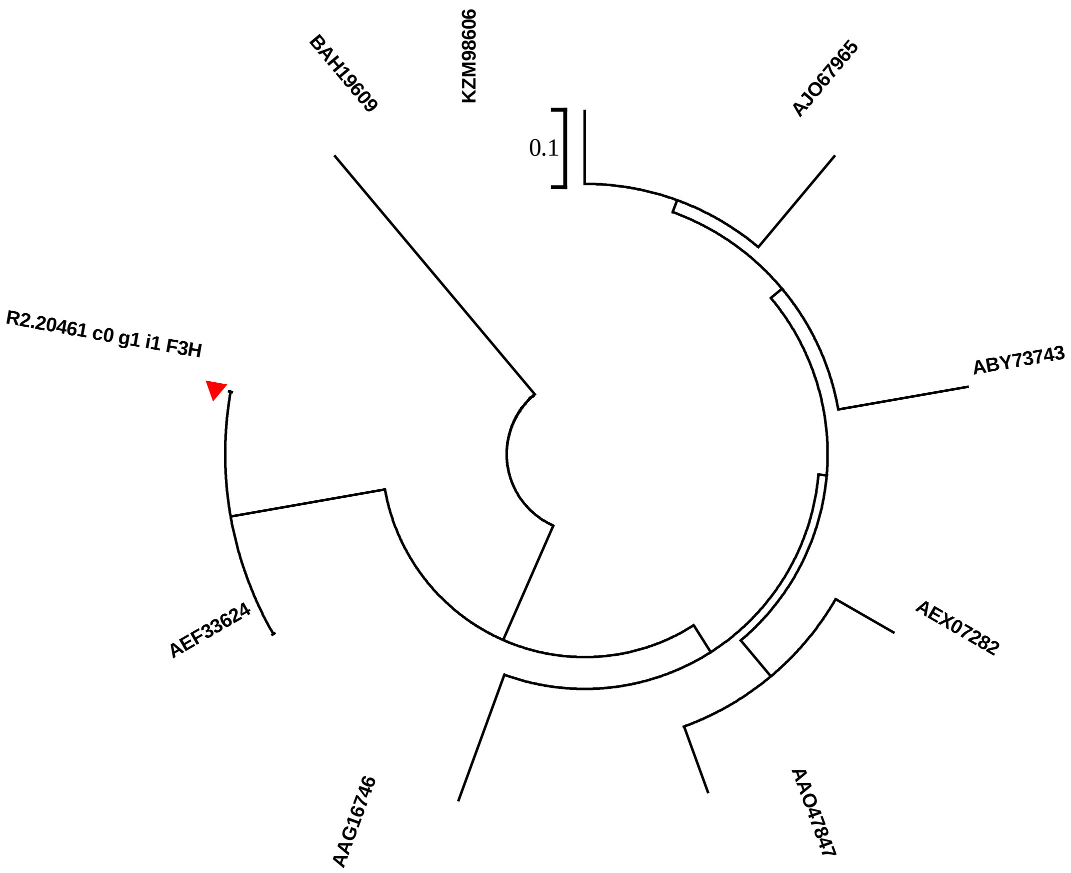

Figure S10. Phylogenetic relationships of the deduced amino acid sequences of one F3′H and other F3′H involved in anthocyanin biosynthesis. The accession numbers of these proteins are as follows: BAH19609: Arabidopsis thaliana/F3'H; AAG16746: Arabidopsis thaliana/F3'H; AEX07282: Arachis hypogaea/F3'H; KZM98606: Daucus carota/F3'H; AAO47847: Glycine max/F3'H; ABY73743: Helianthus annuus/F3'H; AJO67965: Prunus avium/F3'H; AEF33624: Zea mays/F3'H.

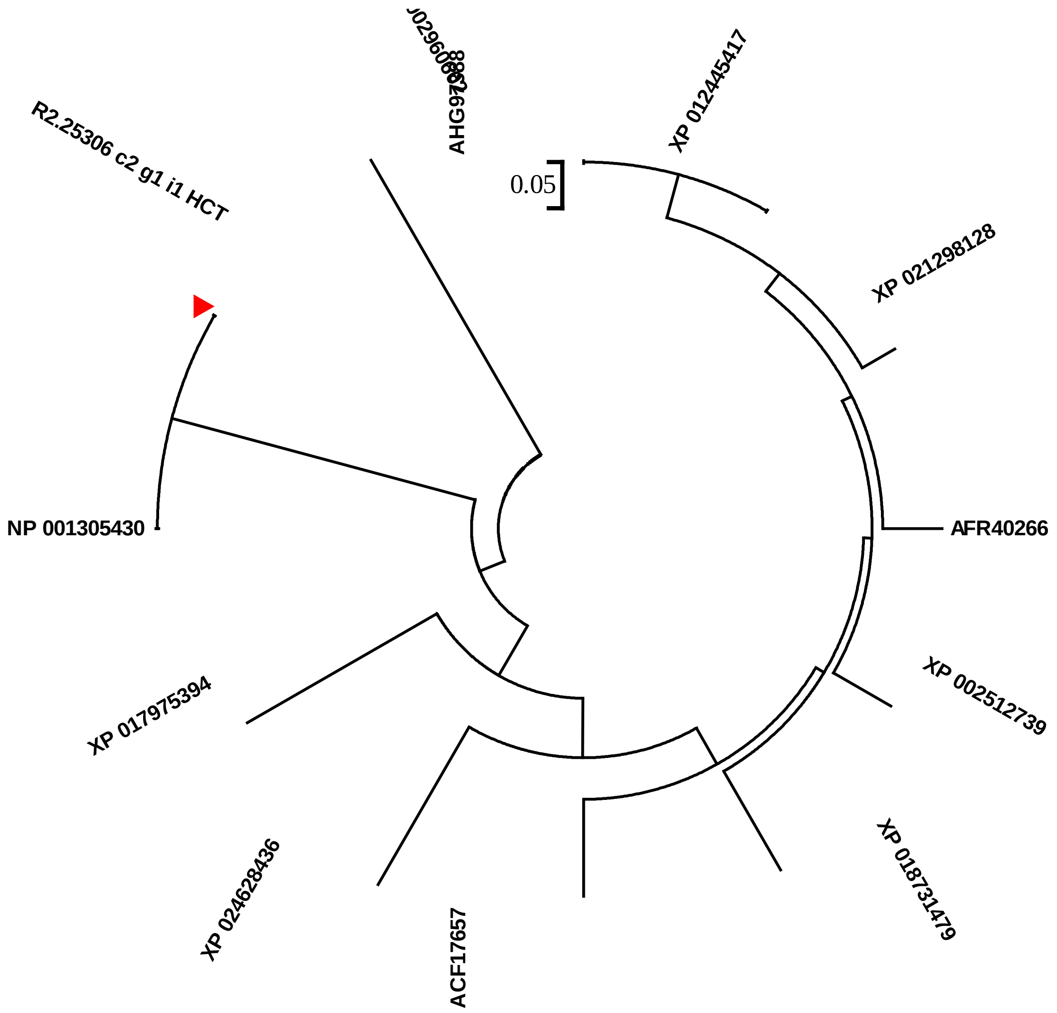

Figure S11. Phylogenetic relationships of the deduced amino acid sequences of one HCT and other HCT involved in anthocyanin biosynthesis. The accession numbers of these proteins are as follows: ACF17657: Capsicum annuum/ HCT; XP_018731479: Eucalyptus grandis/ HCT; AHG97388: Gossypium hirsutum/ HCT; XP_012445417: Gossypium raimondii/ HCT; XP_021298128: Herrania umbratica/ HCT; XP_024628436: Medicago truncatula/HCT; AFR40266: Ricinus communis/HCT; XP_002960602: Selaginella moellendorffii/HCT; XP_017975394: Theobroma cacao/HCT; NP_001305430: Zea mays/ HCT; XP_002512739: Ricinus communis/HCT.

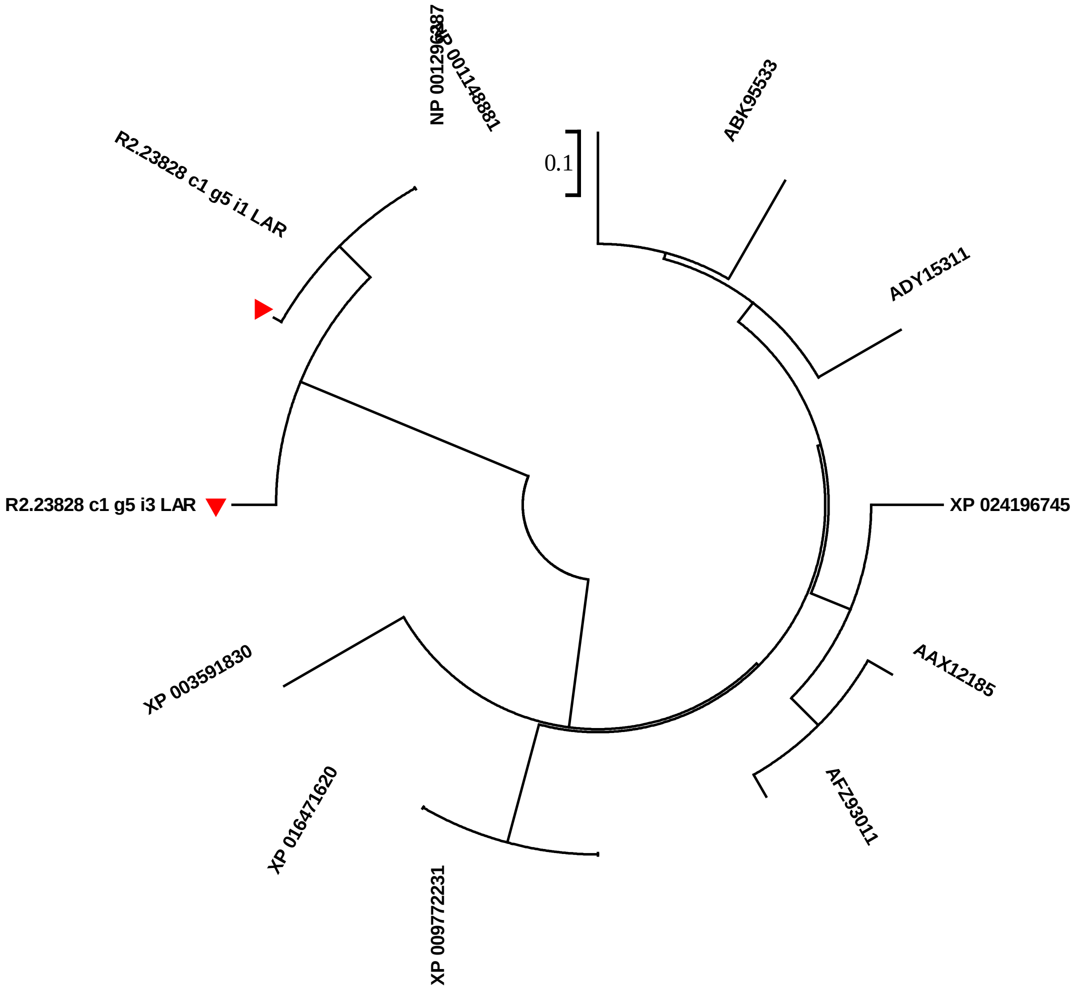

Figure S12. Phylogenetic relationships of the deduced amino acid sequences of two LAR and other LAR involved in anthocyanin biosynthesis. The accession numbers of these proteins are as follows: NP_001296287: Gossypium raimondii/LAR; AAX12185: Malus domestica/LAR; AFZ93011: Malus domestica/LAR; XP_003591830: Medicago truncatula/LAR; XP_009772231: Nicotiana sylvestris/LAR; XP_016471620: Nicotiana tabacum/LAR; ABK95533: Populus trichocarpa/LAR; ADY15311: Prunus avium/LAR; XP_024196745: Rosa chinensis/LAR; NP_001148881: Vitis vinifera/LAR.

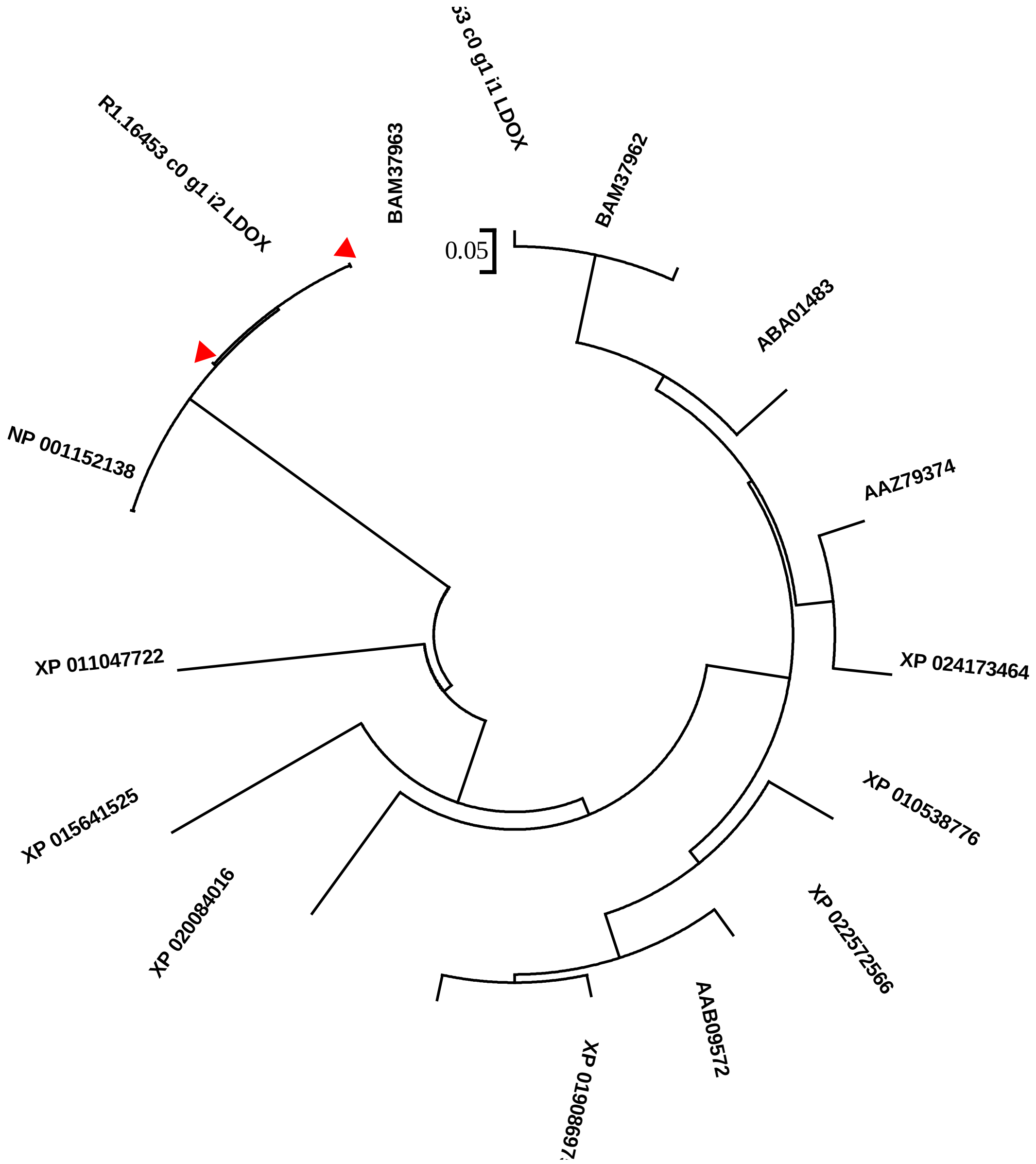

Figure S13. Phylogenetic relationships of the deduced amino acid sequences of two LDOX and other LDOX involved in anthocyanin biosynthesis. The accession numbers of these proteins are as follows: XP_020084016: Ananas comosus/LDOX; AAB09572: Arabidopsis thaliana/LDOX; XP_022572566: Brassica napus/LDOX; XP_019086975: Camelina sativa/LDOX; ABA01483: Gossypium hirsutum/LDOX; AAZ79374: Malus domestica/LDOX; BAM37963: Nicotiana tabacum/LDOX; BAM37962: Nicotiana tabacum/LDOX; XP_015641525: Oryza sativa/LDOX; XP_011047722: Populus euphratica/LDOX; XP_024173464: Rosa chinensis/LDOX; XP_010538776: Tarenaya hassleriana/LDOX; NP_001152138: Zea mays/LDOX.

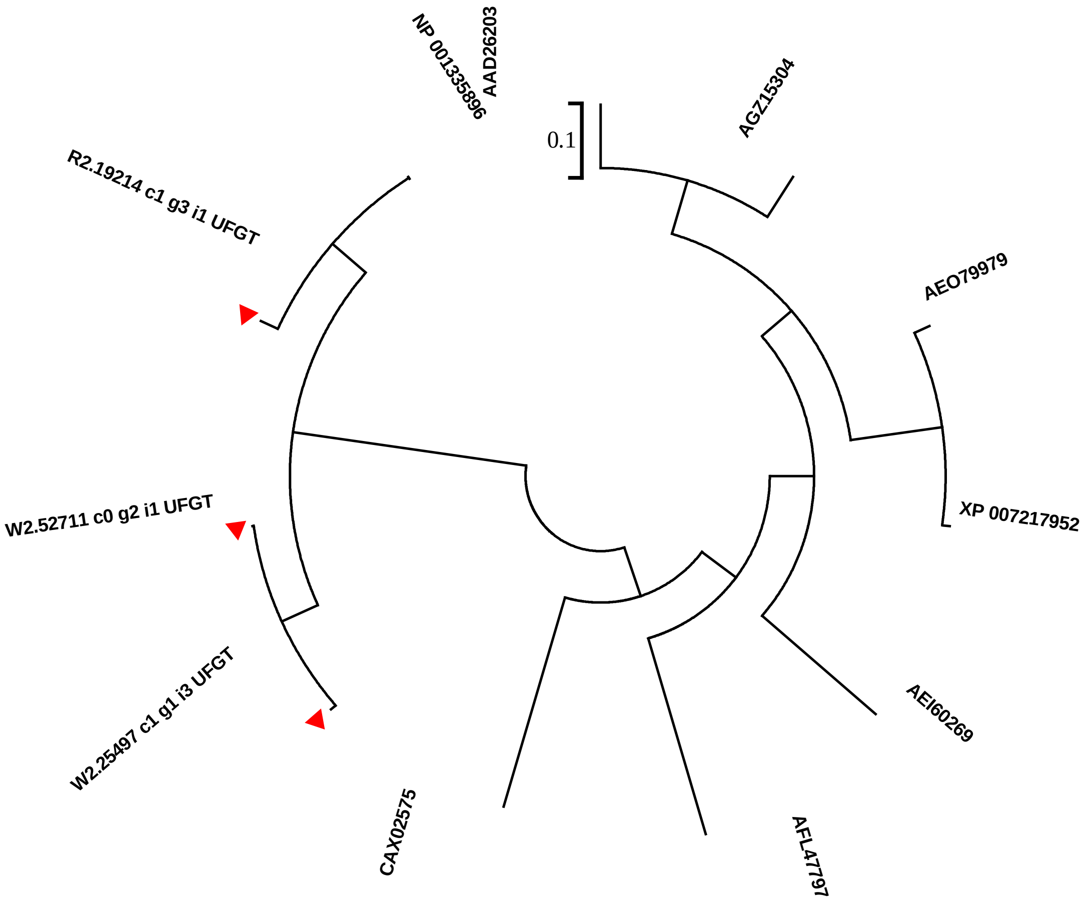

Figure S14. Phylogenetic relationships of the deduced amino acid sequences of three UFGT and other UFGT involved in anthocyanin biosynthesis. The accession numbers of these proteins are as follows: AFL47797: Capsicum annuum/UFGT; AAD26203: Malus domestica/UFGT; AEO79979: Prunus avium/UFGT; XP_007217952: Prunus persica/UFGT; AGZ15304: Pyrus x bretschneideri/UFGT; AEI60269: Vitis vinifera/UFGT; CAX02575: Zea mays/UFGT; NP_001335896: Zea mays/UFGT.

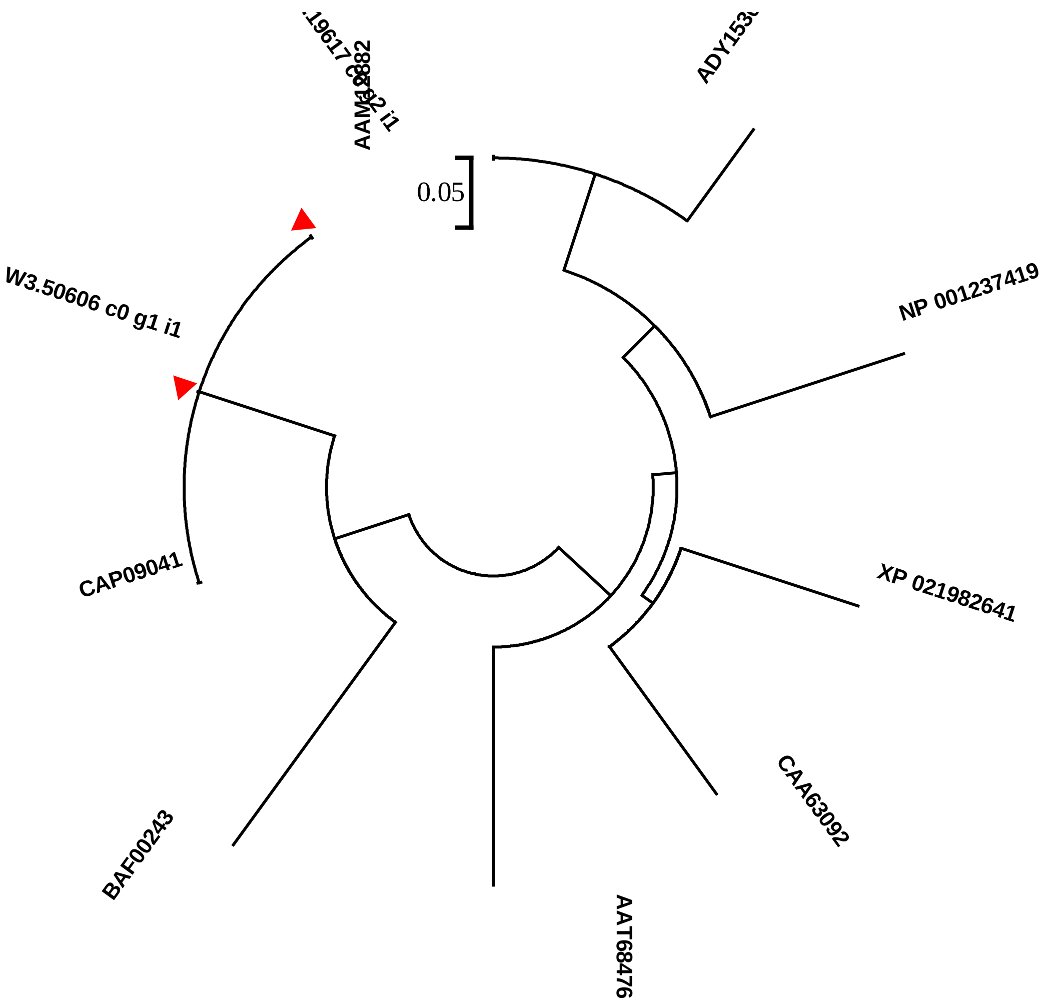

Figure S15. Phylogenetic relationships of the deduced amino acid sequences of two FLS and other FLS involved in anthocyanin biosynthesis. The accession numbers of these proteins are as follows: CAP09041: Arabidopsis thaliana/FLS; XP_021982641: Helianthus annuus/FLS; AAM12882: Malus domestica/FLS; ADY15309: Prunus avium/FLS; CAA63092: Solanum tuberosum/FLS; BAF00243: Arabidopsis thaliana/FLS; NP_001237419: Glycine max/FLS; AAT68476: Allium cepa/FLS.

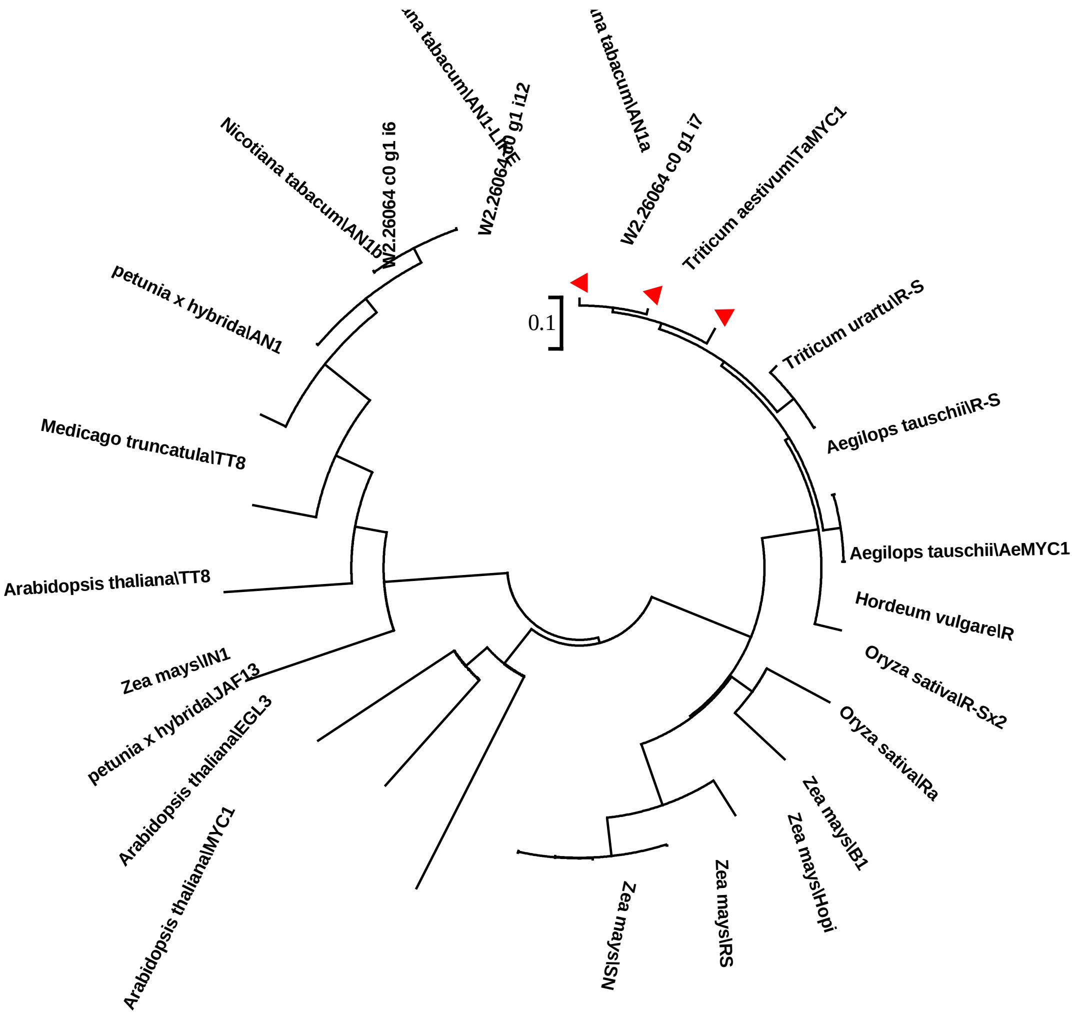

Figure S16. Phylogenetic relationships of the deduced amino acid sequences of 3 MYCs and other MYCs involved in anthocyanin biosynthesis. The accession numbers of these proteins are as follows: AJG36537.1: *Triticum aestivum*/TaMYC1; EMS65005.1: Triticum urartu/R-S; AQM40230: *Hordeum vulgare*/R; XP_020177997.1: *Aegilops tauschii*/R-S; AUK50718.1: *Aegilops tauschii*/AeMYC1; KC771884.1: *Zea mays*/B1; XP_008669036: *Zea mays*/R-S; CAB92300: *Zea mays*/Hopi; NP_001105339: *Zea mays*/SN; XP_006653664: *Oryza sativa*/R-Sx2; AAC49219: *Oryza sativa*/Ra; NP_191957: *Arabidopsis thaliana*/MYC1; NP_176552: *Arabidopsis thaliana*/EGL3; AAC39455: *Petunia x hybrida*/ JAF13; AAB03841: *Zea mays*/IN1; CAC14865: *Arabidopsis thaliana*/TT8; AF260918.1: *Medicago truncatula*/TT8; AF260918.1: *Petunia x hybrida*/AN1; HQ589209.1: *Nicotiana tabacum*/AN1b; NM_001302566.1: *Nicotiana tabacum*/AN1-like; AEE99257: *Nicotiana tabacum*/AN1a.

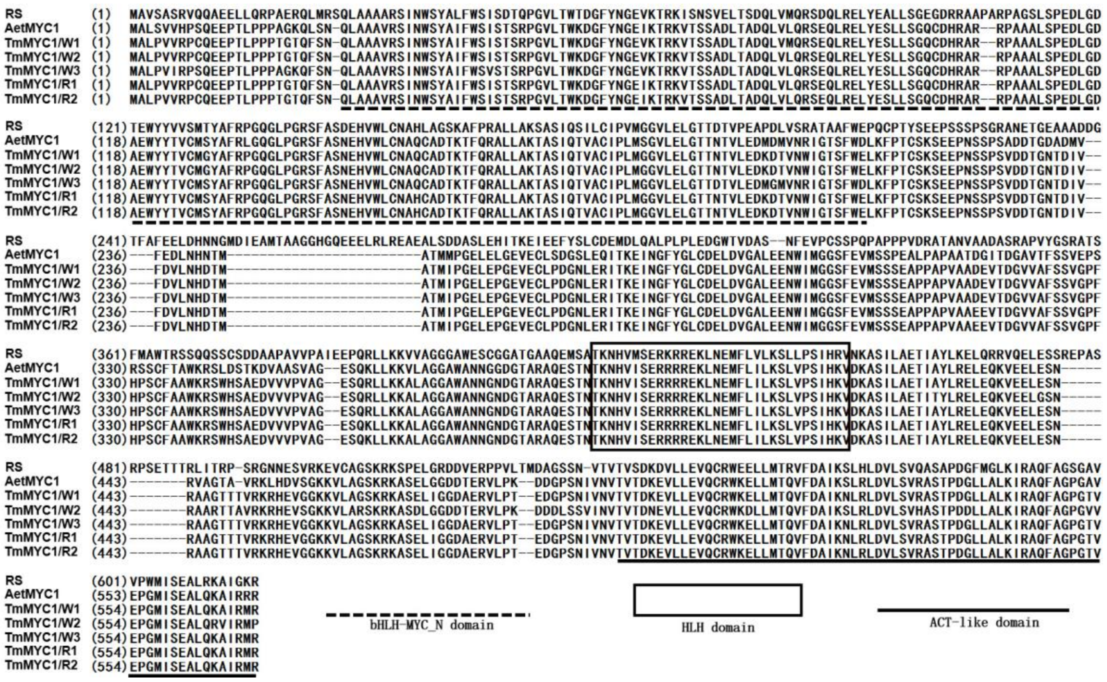

Figure S17. Amino acid sequence alignment of MYC transcription factors from R1, R2, W1, W2, W3 and other species. The black dotted lines represent the conserved bHLH-MYC_N domain, the black rectangle represents theHLH domain, and the solid lines represent the ACT-like domain.

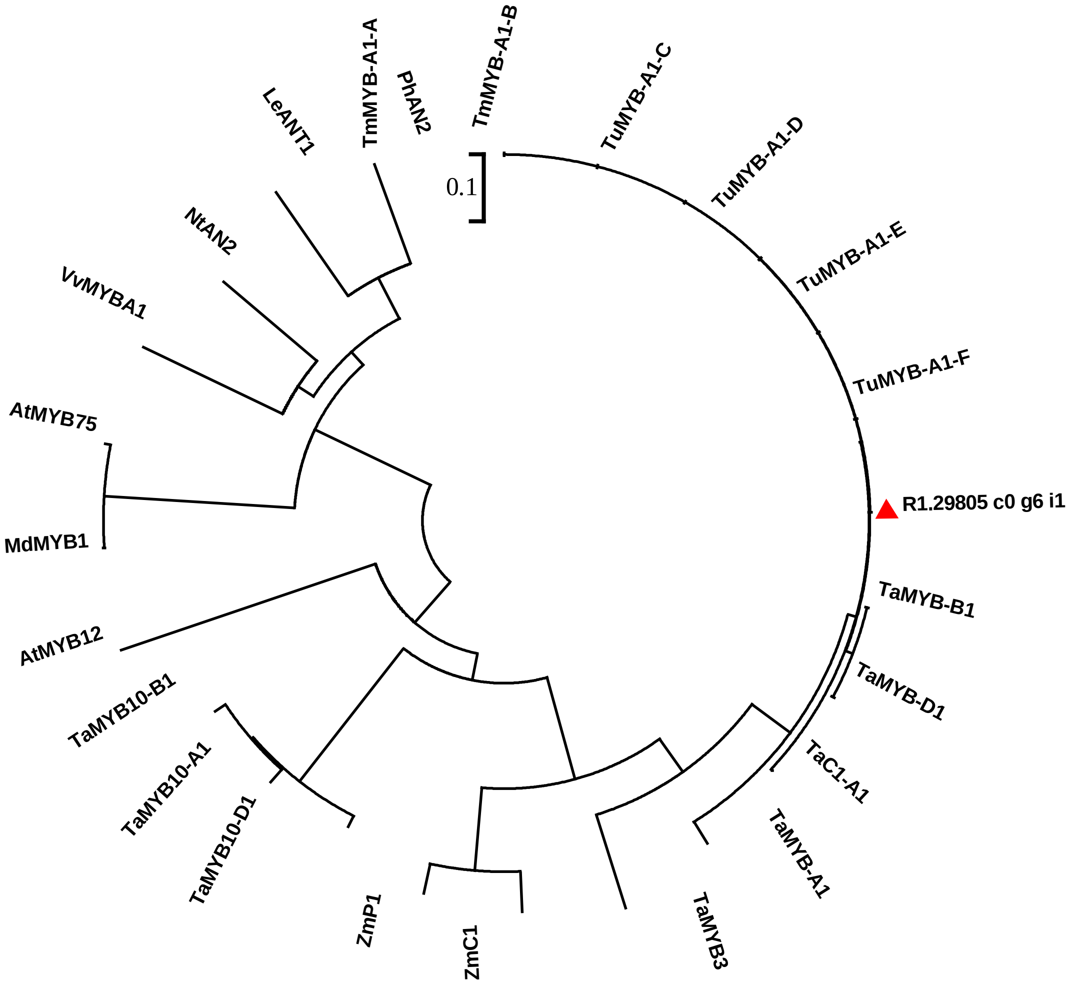

Figure S18. Phylogenetic relationships between MYBs from *T. monococcum* and other species. The accession numbers of these proteins are as follows: ATO60871.1: *Triticum monococcum*/TmMYB-A1-A; ATO60872.1: *Triticum monococcum*/TmMYB-A1-B; ATO60873.1: *Triticum monococcum*/TmMYB-A1-C; ATO60874.1: *Triticum monococcum*/TmMYB-A1-D; ATO60875.1: *Triticum monococcum*/TmMYB-A1-E; ATO60876.1: *Triticum monococcum*/TmMYB-A1-F; AQM56964.1: Triticum aestivum/TaMYB-A1; : Triticum aestivum/TaMYB-B1; AJU57240.1: Triticum aestivum/TaMYB-D1; : Triticum aestivum/TaC1-A1; AB599721: Triticum aestivum/TaMYB10-A1; B599722: Triticum aestivum/TaMYB10-B1; AB191460: Triticum aestivum/TaMYB10-D1; ARB66059.1: Triticum aestivum/TaMYB3; ABB03913: Arabidopsis thaliana/AtMYB12; AAQ55181: Solanum lycopersicum/LeANT1; ABK58136: Malus domestica/MdMYB1; AAF66727: Petunia x hybrida/PhAN2; BAD18977: Vitis vinifera/VvMYBA1; AAA33482: Zea mays/ZmC1; AAA19821: Zea mays/ZmPl; NP_176057.1: Arabidopsis thaliana /AtMYB75; ACO52472.1: Nicotiana tabacum/NtAN2.

Table S1. The information of *T. monococcum* used in this study

Accession ID	Fulltaxa	Latitude	Longitude	Elevation	Country	Coleoptile color
CItr13961	Triticum monococcum L. subsp. monococcum	47.00	-120.00	174.32	USA	White
CItr13962	Triticum monococcum L. subsp. monococcum	47.00	-120.00	174.32	USA	White
PI190939	Triticum monococcum L. subsp. monococcum	-	-	-	Spain	White
CItr13964	Triticum monococcum L. subsp. monococcum	47.00	-120.00	174.32	USA	White
CItr13965	Triticum monococcum L. subsp. monococcum	44.00	-120.00	174.32	USA	White
CItr17659	Triticum monococcum L. subsp. monococcum	-	-	-	-	White
CItr17660	Triticum monococcum L. subsp. monococcum	-	-	-	-	White
CItr17662	Triticum monococcum L. subsp. monococcum	-	-	-	-	White
PI94743	Triticum monococcum L. subsp. monococcum	57.95	38.40	121	Russia	White
PI168806	Triticum monococcum L. subsp. monococcum	38.67	-98.00	466.31	USA	White
PI190945	Triticum monococcum L. subsp. monococcum	-	-	-	-	White
PI190947	Triticum monococcum L. subsp. monococcum	-	-	-	-	White
PI191096	Triticum monococcum L. subsp. monococcum	37.67	-7.00	113.3	Spain	White
PI191097	Triticum monococcum L. subsp. monococcum	36.50	-5.75	201.86	Spain	White
PI191098	Triticum monococcum L. subsp. monococcum	37.72	-3.97	710	Spain	White
PI191146	Triticum monococcum L. subsp. monococcum	-	-	-	Spain	White
PI221329	Triticum monococcum L. subsp. monococcum	-	-	-	Serbia	White
PI265008	Triticum monococcum L. subsp. monococcum	43.20	17.57	177	-	White
PI266844	Triticum monococcum L. subsp. monococcum	53.00	-2.00	181.38	-	White
PI277135	Triticum monococcum L. subsp. monococcum	41.00	20.75	689	Albania	White
PI289599	Triticum monococcum L. subsp. monococcum	-	-	-	-	White
PI289605	Triticum monococcum L. subsp. monococcum	-	-	-	-	White
PI295058	Triticum monococcum L. subsp. monococcum	-	-	-	Bulgaria	White

PI272556	Triticum monococcum L. subsp. aegilopoides (Link) Thell.	47.42	19.33	127.05	Hungary	White
PI306526	Triticum monococcum L. subsp. aegilopoides (Link) Thell.	-	-	-	Romania	White
PI352505	Triticum monococcum L. subsp. aegilopoides (Link) Thell.	-	-	-	-	White
PI355453	Triticum monococcum L. subsp. aegilopoides (Link) Thell.	-	-	-	-	White
PI427447	Triticum monococcum L. subsp. aegilopoides (Link) Thell.	-	-	-	-	White
PI427462	Triticum monococcum L. subsp. aegilopoides (Link) Thell.	-	-	-	-	White
PI487249	Triticum monococcum L. subsp. aegilopoides (Link) Thell.	37.21	42.18	500	Syria	White
PI119423	Triticum monococcum L. subsp. monococcum	39.95	28.85	372	Turkey	White
PI428167	Triticum monococcum L. subsp. monococcum	40.22	29.87	596	Turkey	White
PI272535	Triticum monococcum L. subsp. monococcum	47.42	19.33	128	Hungary	White
PI272561	Triticum monococcum L. subsp. monococcum	47.42	19.33	128	Hungary	White
PI290511	Triticum monococcum L. subsp. monococcum	47.42	19.33	128	Hungary	White
PI418582	Triticum monococcum L. subsp. monococcum	42.00	43.50	1023.19	Azerbaijan	White
PI428166	Triticum monococcum L. subsp. monococcum	40.21	29.19	141	Turkey	White
KT3-2	T.monococcum variet vulgare	-	-	-	-	White
KT3-3	T.monococcum variet flavescens	-	-	-	-	White
KT3-038	T.monococcum strain KUS 68	-	-	-	-	White
KT3-041	T.monococcum strain KUS 155	-	-	-	-	White
KT3-044	T.monococcum strain KUS 282	-	-	-	-	White
PI538724	Triticum monococcum L. subsp. Aegilopoides	37.21	40.10	600	Turkey	White
PI554495	Triticum monococcum L. subsp. monococcum	37.57	38.93	600	Turkey	Red
PI554500	Triticum monococcum L. subsp. monococcum	37.22	39.55	550	Turkey	Red
CItr17657	Triticum monococcum L. subsp. monococcum	-	-	-	-	Red
CItr17658	Triticum monococcum L. subsp. monococcum	-	-	-	-	Red
PI119422	Triticum monococcum L. subsp. monococcum	40.19	29.06	258	Turkey	Red
PI119435	Triticum monococcum L. subsp. monococcum	41.20	36.18	631	Turkey	Red
PI167591	Triticum monococcum L. subsp. monococcum	40.03	27.05	77	Turkey	Red
PI167611	Triticum monococcum L. subsp. monococcum	40.35	27.98	37	Turkey	Red

PI167625	Triticum monococcum L. subsp. monococcum	40.35	27.98	37	Turkey	Red
PI170196	Triticum monococcum L. subsp. monococcum	41.43	27.09	51	Turkey	Red
PI191381	Triticum monococcum L. subsp. monococcum	-	-	-	Ethiopian	Red
PI191383	Triticum monococcum L. subsp. monococcum	-	-	-	Ethiopian	Red
PI221393	Triticum monococcum L. subsp. monococcum	-	-	-	Serbia	Red
PI264935	Triticum monococcum L. subsp. monococcum	35.03	25.00	146	Greece	Red
PI272560	Triticum monococcum L. subsp. monococcum	47.42	19.33	127.05	Hungary	Red
PI277131	Triticum monococcum L. subsp. monococcum	40.31	20.19	206	Albania	Red
PI277136	Triticum monococcum L. subsp. monococcum	41.00	20.75	689	Albania	Red
PI277140	Triticum monococcum L. subsp. monococcum	-	-	-	-	Red
PI286068	Triticum monococcum L. subsp. monococcum	-	-	-	Poland	Red
CItr17673	Triticum monococcum L. subsp. aegilopoides (Link) Thell.	36.40	44.20	1100	Iraq	Red
CItr17741	Triticum monococcum L. subsp. aegilopoides (Link) Thell.	-	-	-	-	Red
PI167627	Triticum monococcum L. subsp. monococcum	40.17	29.08	535.02	Turkey	Red
PI272520	Triticum monococcum L. subsp. aegilopoides (Link) Thell.	47.42	19.33	127.05	Hungary	Red
PI277123	Triticum monococcum L. subsp. aegilopoides (Link) Thell.	-	-	-	-	Red
PI352269	Triticum monococcum L. subsp. aegilopoides (Link) Thell.	-	-	-	-	Red
PI352271	Triticum monococcum L. subsp. aegilopoides (Link) Thell.	-	-	-	-	Red
PI352504	Triticum monococcum L. subsp. aegilopoides (Link) Thell.	-	-	-	-	Red
PI381063	Triticum monococcum L. subsp. aegilopoides (Link) Thell.	34.35	47.23	1600	Iran	Red
PI401412	Triticum monococcum L. subsp. aegilopoides (Link) Thell.	34.35	47.23	1700	Iran	Red
PI401416	Triticum monococcum L. subsp. aegilopoides (Link) Thell.	33.23	48.57	1600	Iran	Red
PI427465	Triticum monococcum L. subsp. aegilopoides (Link) Thell.	-	-	-	Armenia	Red
PI427467	Triticum monococcum L. subsp. aegilopoides (Link) Thell.	39.25	45.50	994.26	Azerbaijan	Red
PI427469	Triticum monococcum L. subsp. aegilopoides (Link) Thell.	39.25	45.50	994.26	Azerbaijan	Red
PI427640	Triticum monococcum L. subsp. aegilopoides (Link) Thell.	36.40	44.20	1100	Iraq	Red
PI427650	Triticum monococcum L. subsp. aegilopoides (Link) Thell.	36.40	44.20	1100	Iraq	Red
PI427720	Triticum monococcum L. subsp. aegilopoides (Link) Thell.	36.92	43.03	750	Iraq	Red

PI427760	Triticum monococcum L. subsp. aegilopoides (Link) Thell.	36.97	43.18	898	Iraq	Red
PI427788	Triticum monococcum L. subsp. aegilopoides (Link) Thell.	34.43	47.43	1350	Iran	Red
PI427796	Triticum monococcum L. subsp. aegilopoides (Link) Thell.	34.83	47.17	1900	Iran	Red
PI427804	Triticum monococcum L. subsp. aegilopoides (Link) Thell.	34.38	47.03	1400	Iran	Red
PI427810	Triticum monococcum L. subsp. aegilopoides (Link) Thell.	34.30	46.18	1513	Iran	Red
PI427818	Triticum monococcum L. subsp. aegilopoides (Link) Thell.	36.40	44.20	1100	Iraq	Red
PI427829	Triticum monococcum L. subsp. aegilopoides (Link) Thell.	36.40	44.20	1100	Iraq	Red
PI427839	Triticum monococcum L. subsp. aegilopoides (Link) Thell.	36.40	44.23	1100	Iraq	Red
PI427844	Triticum monococcum L. subsp. aegilopoides (Link) Thell.	36.42	44.37	1000	Iraq	Red
PI427851	Triticum monococcum L. subsp. aegilopoides (Link) Thell.	36.43	44.38	1000	Iraq	Red
PI427854	Triticum monococcum L. subsp. aegilopoides (Link) Thell.	36.42	44.37	1000	Iraq	Red
PI427859	Triticum monococcum L. subsp. aegilopoides (Link) Thell.	36.42	44.37	1000	Iraq	Red
PI427864	Triticum monococcum L. subsp. aegilopoides (Link) Thell.	36.38	44.23	1000	Iraq	Red
PI427884	Triticum monococcum L. subsp. aegilopoides (Link) Thell.	36.92	43.03	750	Iraq	Red
PI427990	Triticum monococcum L. subsp. aegilopoides (Link) Thell.	33.52	35.87	1141	Lebanon	Red
PI427995	Triticum monococcum L. subsp. aegilopoides (Link) Thell.	33.52	35.87	1141	Lebanon	Red
PI427996	Triticum monococcum L. subsp. aegilopoides (Link) Thell.	33.52	35.87	1141	Lebanon	Red
CItr17674	Triticum monococcum L. subsp. aegilopoides (Link) Thell.	34.43	47.43	1350	Iran	Red
PI427444	Triticum monococcum L. subsp. aegilopoides (Link) Thell.	37.37	36.67	1434	Turkey	Red
PI427622	Triticum monococcum L. subsp. aegilopoides (Link) Thell.	37.78	39.77	1400	Turkey	Red
PI427484	Triticum monococcum L. subsp. aegilopoides (Link) Thell.	37.22	40.12	600	Turkey	Red
PI427509	Triticum monococcum L. subsp. aegilopoides (Link) Thell.	37.23	39.72	670	Turkey	Red
PI427518	Triticum monococcum L. subsp. aegilopoides (Link) Thell.	37.23	39.68	670	Turkey	Red
PI427541	Triticum monococcum L. subsp. aegilopoides (Link) Thell.	37.12	41.03	650	Turkey	Red
PI427542	Triticum monococcum L. subsp. aegilopoides (Link) Thell.	37.12	41.03	650	Turkey	Red
PI427546	Triticum monococcum L. subsp. aegilopoides (Link) Thell.	37.12	41.72	750	Turkey	Red
PI427569	Triticum monococcum L. subsp. aegilopoides (Link) Thell.	37.17	39.03	760	Turkey	Red
PI427600	Triticum monococcum L. subsp. aegilopoides (Link) Thell.	37.23	39.38	688	Turkey	Red

PI427726	Triticum monococcum L. subsp. aegilopoides (Link) Thell.	36.97	43.18	950	Iraq	Red
PI427737	Triticum monococcum L. subsp. aegilopoides (Link) Thell.	36.97	43.18	1250	Iraq	Red
PI427920	Triticum monococcum L. subsp. aegilopoides (Link) Thell.	36.98	43.20	1150	Iraq	Red
PI427746	Triticum monococcum L. subsp. aegilopoides (Link) Thell.	36.97	43.18	898	Iraq	Red
PI554568	Triticum monococcum L. subsp. aegilopoides (Link) Thell.	38.37	37.85	1700	Turkey	Red
PI427972	Triticum monococcum L. subsp. aegilopoides (Link) Thell.	39.32	26.70	30.08	Turkey	Red
PI554516	Triticum monococcum L. subsp. aegilopoides (Link) Thell.	38.60	27.07	30	Turkey	Red
PI428011	Triticum monococcum L. subsp. aegilopoides (Link) Thell.	39.45	47.07	700	Azerbaijan	Red
PI538531	Triticum monococcum L. subsp. aegilopoides (Link) Thell.	37.22	39.78	600	Turkey	Red
PI428168	Triticum monococcum L. subsp. monococcum	40.19	29.06	258	Turkey	Red
PI428169	Triticum monococcum L. subsp. monococcum	40.19	29.06	258	Turkey	Red
PI428164	Triticum monococcum L. subsp. monococcum	40.23	27.24	88	Turkey	Red
PI428175	Triticum monococcum L. subsp. monococcum	40.34	26.69	57	Turkey	Red
PI167589	Triticum monococcum L. subsp. monococcum	40.08	26.83	404.43	Turkey	Red
PI167634	Triticum monococcum L. subsp. monococcum	40.17	28.68	1	Turkey	Red
PI341413	Triticum monococcum L. subsp. monococcum	39.67	31.17	789.54	Turkey	Red
PI167615	Triticum monococcum L. subsp. monococcum	40.05	28.17	24	Turkey	Red
PI428165	Triticum monococcum L. subsp. monococcum	40.10	27.65	45	Turkey	Red
PI554596	Triticum monococcum L. subsp. monococcum	38.60	27.07	30	Turkey	Red
PI237659	Triticum monococcum L. subsp. monococcum	1.00	38.00	696.65	Kenya	Red
PI307984	Triticum monococcum L. subsp. monococcum	35.03	-5.42	457	Morocco	Red
PI343181	Triticum monococcum L. subsp. monococcum	-33.33	-70.67	807.45	Chile	Red
PI345242	Triticum monococcum L. subsp. monococcum	42.00	21.40	255	Macedonia	Red
PI362616	Triticum monococcum L. subsp. monococcum	42.01	21.67	310	Macedonia	Red
PI345133	Triticum monococcum L. subsp. monococcum	43.33	22.13	275	Serbia	Red
PI355546	Triticum monococcum L. subsp. monococcum	47.25	9.92	1179.14	Austria	Red
PI560727	Triticum monococcum L. subsp. monococcum	39.05	41.52	1250	Turkey	Red
PI427959	Triticum monococcum L. subsp. monococcum	36.92	43.03	750	Iraq	Red

PI428151	Triticum monococcum L. subsp. monococcum	42.00	12.50	18.06	Italy	Red
PI428161	Triticum monococcum L. subsp. monococcum	39.79	26.33	101	Turkey	Red
PI428176	Triticum monococcum L. subsp. monococcum	38.18	26.98	192	Turkey	Red
PI435000	Triticum monococcum L. subsp. monococcum	42.55	19.10	72	Montenegro	Red
KT1-1	Triticum monococcum L. subsp. aegilopoides (Link) Thell.	-	-	-	-	Red
KT3-1	Triticum monococcum variet vulgare	-	-	-	-	Red
KT3-5	Triticum monococcum strain vulgare early mutant	-	-	-	-	Red
KT3-4	Triticum monococcum variet hornemanni	-	-	-	-	Red

Table S2. Summary of sequencing data

Samples Name	Raw Reads	Clean Reads	Clean Bases	GC content (%)	Q30 (%)
R1	61,346,032	60,710,038	9,007,221,392	56.51	91.24
R2	54,026,952	53,452,682	7,931,706,181	56.49	90.89
W1	59,678,366	59,050,664	8,764,858,073	56.46	90.81
W2	58,840,658	58,269,140	8,645,739,425	55.83	91.15
W3	58,452,216	57,887,880	8,597,059,382	56.23	91.17

Table S3. The annotation summary of predicted protein numbers from various databases

Values	Total	Nr	Swissprot	KEGG	COG	Overall
Number	294,658	171,946	85,516	48,893	64,605	176,630
Percentage	100%	58.35%	29.02%	16.60%	21.93%	59.94%

Table S4. Information on unigenes associated with anthocyanin biosynthesis in coleoptiles.

Gene	KEGG orthology	KEGG enzyme	Reference genes Length（bp）	geneID	Length(bp)	Chromosome	Expression				
							R1_FPKM	R2_FPKM	W1_FPKM	W2_FPKM	W3_FPKM
PAL	K10775	EC:4.3.1.24	2148	R1.26388_c2_g1_i2	2658	6A	60.57	52.66	0.00	0.10	0.00
				Average			56.62			0.03	
				Times					1698.45		
C4H	K00487	EC:1.14.13.11	1518	R1.25946_c1_g1_i2	2193	3A	71.17	60.00	47.90	38.77	40.50
				R1.25946_c1_g1_i1	1576	3A	42.78	23.28	5.15	5.82	5.99
				In total			113.95	83.28	53.05	44.59	46.49
				Average			98.62			48.04	
				Times					2.05		
4CL	K01904	EC:6.2.1.12	1692	R1.23474_c0_g1_i1	287	2A	1.06	0.00	0.00	0.00	0.00
				R1.23474_c0_g2_i1	2122	2A	5.69	3.85	1.58	1.85	1.17
				R1.23474_c0_g3_i1	563	2A	1.55	0.35	0.29	0.00	0.00
				In total			8.30	4.20	1.87	1.85	1.17
				Average			6.25			1.63	
				Times					3.83		
HCT	K13065	EC 2.3.1.133	1305	R2.25306_c2_g1_i1	1790	3A	1.95	5.87	1.39	1.17	1.01
				Average			3.91			1.19	
				Times					3.29		
CHS	K00660	EC:2.3.1.74	1206	R2.11261_c0_g1_i1	1731	2A	103.87	105.88	74.95	58.96	57.46
				W1.21718_c1_g2_i2	796	2A	8.60	5.40	22.76	9.73	13.16
				W3.16810_c0_g1_i1	471	2A	8.43	4.80	4.62	2.02	2.40
				In total			120.90	116.08	102.33	70.71	73.02

				Average			118.49		82.02	
				Times				1.44		
CHI	K01859	EC:5.5.1.6	684	W3.16226_c0_g3_i1	1244	5A	0.25	0.48	0.00	0.13	0.44
				W3.16226_c0_g2_i1	1158	5A	175.78	150.04	89.62	72.88	106.87
				W1.699_c1_g2_i1	1288	5A	10.72	7.31	9.04	5.84	5.03
				In total			186.75	157.83	98.66	78.85	112.34
				Average			172.29			96.62	
				Times					1.78		
F3H	K00475	EC:1.14.11.9	1077	R2.58152_c0_g1_i1	2246	2A	52.02	34.44	39.25	45.93	42.69
				R2.13996_c1_g1_i1	2489	2A	57.74	36.60	40.41	46.34	41.40
				In total			109.76	71.04	79.66	92.27	84.09
				Average			90.40			85.34	
				Times					1.06		
F3'H	K05280	EC:1.14.13.21	1539	R2.20461_c0_g1_i1	882	2A	1.48	1.42	0.1	0.71	0.11
				Average			1.45			0.31	
				Times					4.73		
F3′5′H	K13083	EC:1.14.13.88	1539	W2.26764_c0_g2_i1	2735	2A	2.81	2.47	1.08	0.99	0.84
				Average			2.64			0.97	
				Times					2.72		
DFR	K13083	EC:1.14.13.88	1074	W2.22235_c0_g2_i3	1422	3A	0.00	7.63	0.27	5.01	2.19
				R1.13446_c0_g1_i1	1435	3A	66.63	38.10	0.00	0.00	0.00
				W2.22235_c0_g2_i2	1495	3A	0.00	0.14	0.00	0.84	0.13
				R1.13446_c0_g2_i1	1507	3A	8.95	4.03	0.00	0.15	0.00
				In total			75.58	49.90	0.27	6.00	2.32
				Average			62.74			2.86	
				Times					21.91		

LDOX	K05277	EC:1.14.11.19	1062	R1.16453_c0_g1_i2	675	5A	4.41	10.51	6.62	2.13	2.82
				R1.16453_c0_g1_i1	579	5A	2.11	1.25	2.24	1.56	0.75
				In total			6.52	11.76	8.86	3.69	3.57
				Average			9.14			5.37	
				Times					1.70		
LAR	K13081	EC 1.17.1.3	1032	R2.23828_c1_g5_i3	2201	5A	11.50	12.89	0.36	0.05	0.00
				R2.23828_c1_g5_i1	1723	5A	31.28	42.49	0.17	0.01	0.49
				In total			42.78	55.38	0.53	0.06	0.49
				Average			49.08			0.36	
				Times					136.33		
ANR	K08695	EC:1.3.1.77	891	W1.24704_c3_g2_i2	1425	2A	0.04	0.06	0.07	0.00	0.08
				R2.23828_c1_g4_i1	424	2A	5.23	3.50	3.55	4.39	3.30
				R2.23828_c1_g5_i3	2201	5A	11.50	12.89	0.36	0.05	0.00
				W3.27164_c4_g1_i2	1332	2A	3.46	2.14	5.39	2.49	3.68
				R2.23828_c1_g4_i2	489	2A	0.20	0.00	0.56	0.72	0.35
				R1.25388_c2_g1_i1	367	2A	0.39	0.00	0.00	0.00	0.00
				R2.67278_c0_g2_i1	334	2A	8.06	0.76	9.29	2.64	18.67
				R2.67278_c0_g1_i1	562	2A	0.12	2.15	0.00	0.13	0.13
				In total			29.00	21.50	19.22	10.42	26.21
				Average			25.25			18.62	
				Times					1.36		
UFGT	K13496	EC:2.4.1	2607	W2.25497_c1_g1_i3	942	7A	2.02	2.50	0.96	7.31	5.70
				W2.52711_c0_g2_i1	552	7A	0.46	0.17	0	9.77	6.19
				R2.19214_c1_g3_i1	1647	7A	66.52	49.35	4.39	1.53	4.82
				In total			69.00	52.02	5.35	18.61	16.71
				Average			57.94			13.56	

			Times					4.27		
MYB	K09422	774	R1.29805_c0_g6_i1	1314	7A	17.64	18.04	1.60	1.38	1.04
			Average			17.84			1.34	
			Times					13.31		
MYC	K13422	1768	W2.26064_c0_g1_i12	2194	2A	5.22	0.31	1.36	5.52	9.15
			W2.26064_c0_g1_i7	2183	2A	2.41	0.00	2.75	2.81	1.75
			W2.26064_c0_g1_i6	1963	2A	7.67	1.11	6.63	6.99	7.65
			In total			7.63	2.65	4.11	8.33	10.9
			Average			5.14			7.78	
